# Modelling nano-particle-PEG-NLS complexes for nucleus-targeted drug delivery: revisiting the multi-dynein nano-cargo transport on microtubules

**DOI:** 10.1080/10717544.2026.2738276

**Published:** 2026-10-12

**Authors:** Tal Yagev, Itay Fayer, Itay Adar, Gal Halbi, Anne Bernheim-Groswasser, Rony Granek

**Affiliations:** a Avram and Stella Goldstein-Goren Department of Biotechnology Engineering, Ben-Gurion University of the Negev, Beer Sheva, Israel; b Department of Chemical Engineering, Ben-Gurion University of the Negev, Beer Sheva, Israel; c Ilse Katz Institute for Nanoscale Science and Technology, Ben-Gurion University of the Negev, Beer Sheva, Israel; d Department of Physics, Ben-Gurion University of the Negev, Beer Sheva, Israel

**Keywords:** Dynein motors, nucleus-targeted, drug delivery, nanoparticles, intracellular active transport, stochastic simulations, microtubule-based transport, semi-flexible polymers

## Abstract

Efficient transport to the nuclear envelope is a prerequisite for many nucleus-targeted drug delivery strategies. We develop an enhanced, energy-based simulation of spherical nanoparticle (NP) complexes hauled by multiple dynein motors via flexible polymer linkers, aimed at deriving design rules for nucleus-directed delivery. The model extends prior work by treating dynein as a semi-flexible polymer and enforcing path-wise steric exclusion along full stepping trajectories. Event propensities follow a Monte-Carlo scheme with energies that couple linker entropy, dynein bending, and binding enthalpy; post-event mechanical relaxation uses gradient descent. We compare our model results to recent motility assays of spherical NPs decorated with polyethylene-glycol (PEG) polymers, each ending with a nuclear-localisation-signal (NLS) peptide that can recruit dynein from the cytoplasm. With standardised biophysical parameters, the model captures the observed dependence on motor number: longitudinal velocity decreases with dynein load, whereas run-distance and run-time increase. For mean NP-bound motors between 1 and 6, the model results lie within the experimental 95% confidence intervals. A systematic scan of polymer contour length, for a 20 nm radius cargo hauled by six motors, reveals a clear optimum of the run-time and run-length at about 40 nm length, despite a reduction in velocity. Similar results, for different cargo sizes and motor loading, can be obtained using our model-based computer code. They can provide a quantitative basis for tuning linker contour length (and, secondarily, motor loading by tuning PEG-NLS density) when engineering NP vehicles that exploit native microtubule transport for nucleus-directed delivery.

## 1 Introduction

The nucleus is a favourite intracellular target for drug or gene delivery by nano-carriers. It encounters a few bottlenecks (Barua and Mitragotri 2014; Nie et al. 2023): (i) arrival to the target tissue, (ii) cellular endocytosis, (iii) endosomal release, (iv) transport to the nuclear envelope, and (v) passage through the nuclear-pore complex. Among those, very few studies focus on enhancing the efficiency of transport towards the nucleus. Use of a nuclear localisation signal (NLS) peptide as a ligand has been suggested (Salman et al. 2005; Lott and Cingolani 2011), due to its ability to recruit the dynein motor protein (following binding of α - and β -importins) that can carry the cargo towards the centrosome. NLS also serves, following its recruitment of β -importin, as a gateway to the nucleus (Lott and Cingolani 2011).

Intracellular transport by microtubule-associated motors is essential for organising eukaryotic cells and positioning cargos over micrometre scales. Kinesin typically drives anterograde motion toward the cell periphery (the plus-end), whereas dynein walks towards the minus-end and transports cargos retrogradely towards the cell centre and nuclear envelope (Hirokawa 1998; Vale 2003; Pilling et al. 2006; Hirokawa et al. 2009). Viruses harness this native machinery, for example via nuclear-localisation motifs and adaptor-mediated dynein recruitment, to reach perinuclear sites (Kelkar et al. 2004; Scherer and Vallee 2011; Jayappa et al. 2012; Campbell and Hope 2015; Dharan and Campbell 2018; Carnes and Aiken 2019). These observations motivate synthetic, nucleus-directed delivery vehicles that exploit dynein for intracellular trafficking (Cohen and Granek 2014; Peker and Granek 2016; Fayer and Granek 2019).

While single-dynein motility is well characterised (Reck-Peterson et al. 2006; Gennerich et al. 2007; Qiu et al. 2012; Can et al. 2019), its limited processivity time ≃1 s and velocity ≃0.8μm/s do not allow it to carry a cargo from the cell periphery to the nuclear envelope in a single run. Nanoparticles (NPs) that engage multiple motors have been shown to overcome this limitation since a single ‘run’ is maintained so long as at least one (cargo) motor is attached to the MT and keeps walking on it. However, optimal engineering of this type of NPs requires a biophysical modelling that captures cooperative effects, steric constraints, and geometry-dependent energy costs (Gross et al. 2007; Müller et al. 2008, Müller et al. 2010; Hendricks et al. 2012; Derr et al. 2012; Cohen and Granek 2014; Hancock 2014; Peker and Granek 2016).

A key design feature is to anchor the NP with a dense array of spacer polymers, such as polyethylene glycol (PEG), each ending with an NLS peptide (Cohen and Granek 2014; Halbi et al. 2021, 2023). Importantly, the design only deals with the decoration of the NP regardless of the material it is made of or the drug it carries, which makes this approach potentially transferable to other solid or solid-like NPs, provided their geometry, surface chemistry, and motor-loading statistics are appropriately specified. The role of the NLS is to recruit dynein from the cytoplasm, which is done in three steps (Halbi et al. 2023): binding of α -importin, then β -importin, and finally dynein. The polymer allows easy stretching of the PEG-NLS- α - β -dynein complex, which is required for binding to the MT of complexes that do not face the MT (Cohen and Granek 2014). Without such a stretch, a multi-motor transport would not be achievable. Recent motility assays of NP constructs by Halbi et al. in HeLa cell cytoplasmic extracts—designed according to this protocol—showed a substantial increase in run-times and run-lengths much above the characteristic single dynein values, confirming the hypothesis of a multi-motor transport (Halbi et al. 2021, 2023). Moreover, Western Blot assays allowed to deduce the number of dyneins that are bound to the NP, either in the original extract or upon its dilutions.

A notable theoretical advance was a 3D model simulation of dynein-driven NP motion developed by Granek and coworkers (Cohen and Granek 2014; Peker and Granek 2016; Fayer and Granek 2019; Halbi et al. 2021, 2023), which couples Gaussian-linker elasticity, spatial motor coordination, excluded volume, and a realistic microtubule (MT) binding-site lattice (Nishinari et al. 2005; Klumpp and Lipowsky 2009). Despite these developments, quantitative gaps between theory and experiment remained (Halbi et al. 2023). In particular, while the diminishing of velocity with motor number rise was observed in both, this effect was underestimated by the model. Similarly, while both run-time and run-length are rising with increasing NP-bound motors, these are also significantly underestimated by the model, which limits the applicability of the model as a precise quantitative tool for cargo optimal design.

Here, we suggest that these discrepancies are associated with the following two assumptions: dynein treated as a rigid body, and excluded volume enforced only at the destination site of a step. To examine this quantitatively, we relax these simplifications. We develop an enhanced, energy-based model and associated Monte Carlo (MC) computer code that introduce two physical upgrades: (i) dynein is modelled as a semi-flexible polymer with worm-like-chain bending energy, allowing deformation to partition between motor bending and linker extension; and (ii) excluded volume is enforced path-wise along full stepping trajectories, eliminating non-physical overlaps during approach to a target site. Event propensities are computed within a MC simulation framework using energies that couple linker entropy, dynein bending, and dynein-MT binding enthalpy; post-event mechanical relaxation uses gradient descent. Code-level streamlining and parallel execution reduce end-to-end wall time from months to <3 h for full sweeps, enabling systematic parameter exploration.

The model-code is run using standardised biophysical parameters and previously published datasets for PEG-linked, NLS-decorated NPs (Halbi et al. 2021, 2023). Using about a thousand trajectories per NP parameter set, the corrected model is shown in this paper to capture the observed motor-number dependencies within the experimental 95% confidence intervals for most tested conditions, rather than claiming uniformly high quantitative accuracy, thus allowing us to deduce design rules for dynein-powered, nucleus-directed delivery. In what follows, we detail the model, validate its predictions against experiment, and derive delivery-relevant trade-offs and parameter sensitivities.

## 2 Materials and methods

### 2.1 System overview

For clarity, here we both repeat the model and simulation algorithm presented in Ref. Halbi et al. (2021) (see Supplementary Material therein) and emphasise the new features that allowed better agreement with experimental results.

Relative to the previous model, we retain the spherical NP geometry, Gaussian PEG-linker entropy, MT lattice representation, detailed-balance attachment/detachment kinetics, and the quasi-equilibrium relaxation framework. The two changes examined here are specifically (i) replacement of the rigid dynein by a semi-flexible worm-like chain and (ii) enforcement of excluded volume along the complete approach path of a stepping motor rather than only at its destination site. Thus, the comparison between the present and previous formulations isolates the effects of dynein compliance and path-wise steric exclusion while retaining the underlying kinetic and geometric framework. We simulate a spherical nanoparticle (NP; radius 20 nm ) propelled along a single microtubule (MT; radius 12.5 nm ) by n dynein motors tethered via flexible PEG linkers. Grafting sites are sampled uniformly over the NP surface (isotropic attachment), and each tether terminates at one motor (Halbi et al. 2021, 2023). The MT is represented as a rigid cylinder whose physical dimensions match the MT lattice (Kon et al. 2012; Hirose 2012). Following our prior framework, the MT surface is effectively discretized into the dynein’s microtubule binding domain (MTBD) target sites; for visualisation and bookkeeping, we unwrap the accessible half-cylinder into a 2D chart with longitudinal coordinate x (protofilament axis; site spacing 8  nm ) and an effective transverse span y of 25 nm (used consistently in earlier work). Sites on the lower half of the cylinder are excluded by steric constraints (Kon et al. 2005; Can et al. 2014).

Dynein is modelled geometrically as a semi-flexible polymer of effective length 60nm , persistence length 2μ m (Kamiya et al. 2016), and diameter 12nm (forbidden radius 12nm ), consistent with reproducing the spatial footprint of the bound motor (Reck-Peterson et al. 2006; Gennerich et al. 2007). PEG linkers are modelled as Gaussian chains with contour length $L_{c}$ and Kuhn length a ; unless otherwise stated, $L_{c}=42.56\;\mathrm{nm}$ and a=0.75nm (De Gennes 1979; Doi and Edwards 1988). All simulations run at T=298K . A schematic is shown in Figure 1.

**Figure 1.**
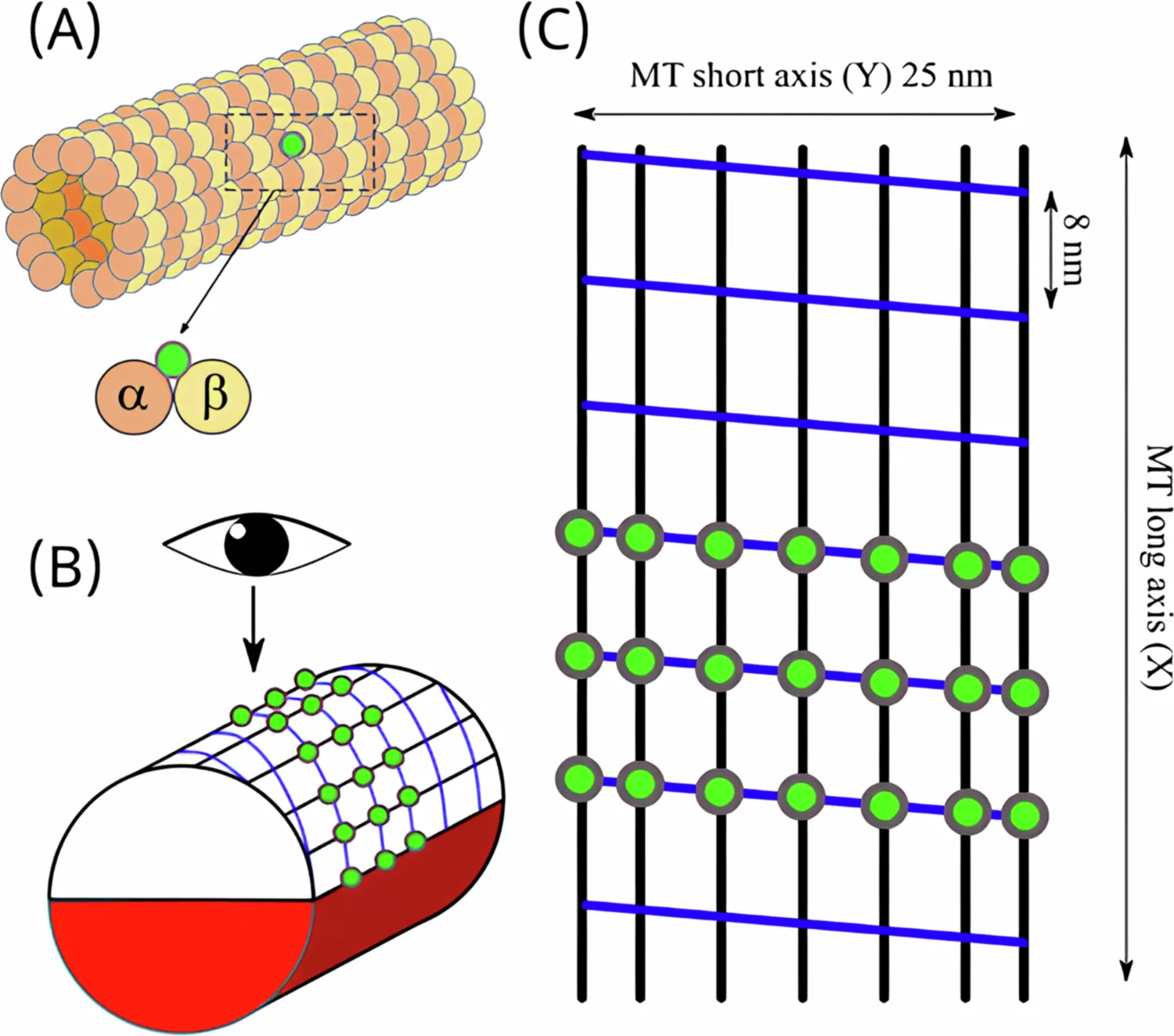
System overview and Microtubule (MT) binding-site lattice. (A) MT built from α -tubulin (orange) and β -tubulin (yellow); a representative dynein MTBD target site is marked (green). (B) The MT is modelled as a rigid cylinder decorated with a discrete network of effective MTBD binding sites (green). The lower half-cylinder (red) is sterically inaccessible and excluded from binding/stepping. (C) Plane-projected map (‘bird’s-eye’) of the accessible half-cylinder used for bookkeeping: longitudinal coordinate x follows protofilaments with site spacing 8nm ; the effective transverse span y is 25nm . Green dots denote allowed sites; blue slanted lines indicate transverse diagonals spaced 8nm ; vertical black lines correspond to protofilaments. This lattice defines candidate attach/step locations and the geometry used in the path-wise steric check. Schematic; not to scale.

#### 2.1.1 Parameter summary for reproducibility

Table 1 lists the fixed physical parameters used throughout; figure captions state any deviations or scanned ranges.

**Table 1.** Physical parameters used in simulations.

Quantity	Value (default)
NP radius $R_{\mathrm{NP}}$	20 nm
MT radius $R_{\mathrm{MT}}$	12.5 nm
Dynein effective length	60 nm
Dynein forbidden radius	12 nm
PEG contour length $L_{c}$	42.56 nm
PEG Kuhn length a	0.75 nm
Dynein persistence length $L_{p}$	2 µm
Temperature T	298 K
Effective temperature $T_{\mathrm{eff}}$	1900 K
Single-motor detachment baseline $k_{0}$	$1\;s^{-1}$
MT longitudinal site spacing	8 nm

### 2.2 Motor dynamics model and kinetic Monte Carlo algorithm

Each trajectory begins when the first motor attaches to the MT and ends when the last bound motor detaches (the NP run terminates) (Halbi et al. 2021, 2023). System evolution is simulated with a variable time-step kinetic MC algorithm—bearing some similarities to the Gillespie stochastic simulation algorithm (GSSA) (Gillespie 1977)—over a discrete event set (Figure 3A):

1. Attachment of a free motor to an available MT site,
2. Detachment of a bound motor,
3. A stepping event by a bound motor along the MT surface,
4. A ‘jamming’/null-steric event: a proposed attach/step would violate excluded volume and is rejected (Vilfan 2005; Guérin et al. 2010).

Let $k_{j}$ denote the propensity (rate) of event j in the current configuration and define $k_{\mathrm{tot}}=\sum_{i}k_{i}$ . We sample the event index with probability

$$P_{j}=\frac{k_{j}}{k_{\mathrm{tot}}},$$

and advance the simulation clock using the variable MC time-step,

$$\delta t=\frac{1}{k_{\mathrm{tot}}},$$

For efficiency, events are grouped into attachment, detachment, and stepping classes; letting $K_{a}=\sum_{j\in a}k_{j}$ , we first draw a class with $P\left(a\right)=K_{a}/k_{\mathrm{tot}}$ and then an event within that class with $P\left(j|a\right)=k_{j}/K_{a}$ . After executing the event, all propensities are updated and time is advanced by δt . Importantly, except for jamming events, with this choice of δt , there is no loss of MC time on MC steps where no configurational changes occur.

**Path-wise steric exclusion.** Excluded-volume constraints are enforced along the entire approach path of a proposed attachment or step. Here, we improved on the previous algorithm (Halbi et al. 2023) that accounted only for the excluded-volume constraints in the target location. Thus, a trial motor stepping is admissible only if the swept volume of the moving motor does not intersect the forbidden volume of any already-bound motor (see Supplemental data Section S5; Figures S3 and S4) (Vilfan 2005; Guérin et al. 2010).

**Null steric/** **‘jamming’ events.** To preserve the attempt-process timescale, trials that fail the path-wise steric (‘excluded volume’) prohibition are recorded as jamming events, which still advance simulation time by δt ; the configuration remains unchanged. This bookkeeping preserves detailed balance and waiting times governed by the total attempt rate (Seifert 2012).

Following each stochastic event, the system is rapidly relaxed to a local free-energy minimum by (i) translating the NP along the MT (longitudinal, lateral, radial), (ii) rotating it about its axis, and (iii) re-optimising the dynein bending conformations (semi-flexible motors), yielding a stretched-to-relaxed transition (Figure 3B). The minimised free energy includes linker entropic elasticity, dynein bending energy, and motor–MT binding enthalpy (Halbi et al. 2021, 2023). This quasi-equilibrium relaxation is treated as instantaneous relative to δt (see Quasi-equilibrium section).

### 2.3 Model energy calculations

Each dynein is connected to the NP via a flexible PEG linker; the configuration of each motor–polymer pair is determined by minimising the total mechanical energy at attachment and stepping events. We assume the grafting density lies in the ‘mushroom’ regime, neglecting polymer–polymer excluded volume (De Gennes 1979). The ‘mushroom’ approximation concerns the PEG tethers themselves and should not be identified with the number of dyneins recruited to a particle. The simulations explicitly account for steric exclusion between motor bodies bound to the MT and along their stepping paths, while PEG–PEG excluded-volume interactions are neglected as in the preceding model. For the 5 kDa PEG used experimentally, the estimated radius of gyration is only about 2.3 nm (Supplemental Section S1). With an NP radius of 20 nm and a simulated mean motor load of at most 13, this corresponds to a sparse motor occupancy on the NP surface with PEG surface density ~ 0.003 nm^−2^, which is still one order of magnitude below the overlap threshold ~ 0.2 nm^−2^. At higher motor loadings, the dominant additional crowding effect occurs on the MT surface and is treated explicitly through the motor forbidden-volume construction. The total free energy contribution of a single connected motor combines linker entropy, dynein bending, and binding enthalpy:

(1) $$\Delta U_{i}=\Delta U_{\text{linker},i}+\Delta U_{\text{bend},i}-\epsilon ,$$

where the binding enthalpy, i.e. the energy associated with attachment of a dynein MTBD to an MT site, is $\epsilon \approx 8k_{B}T$ (Cohen and Granek 2014).

The total free energy of the system is given as the sum of the individual motor contributions:

(2) $$\Delta U_{\text{system}}=\sum_{i=1}^{n}\Delta U_{i}.$$

#### 2.3.1 Linker entropy

An end of a tethered Gaussian chain, which is anchored on the NP surface at the origin, is described in local cylindrical coordinates (ρ,z) . The entropic stretching cost is (Doi and Edwards 1988; Rubinstein and Colby 2003)

(3) $$\Delta U_{\text{linker}}=-k_{B}T\left[\ln\left(\frac{9za^{3}}{2\pi R_{0}^{4}}\right)-\frac{3\left(\rho^{2}+z^{2}\right)}{2R_{0}^{2}}\right],$$

with Kuhn length a=0.75nm and $R_{0}$ the RMS end-to-end distance for contour length $L_{c}$ (coordinate transforms in Supplemental S4; see Figure 2A,B).

**Figure 2.**
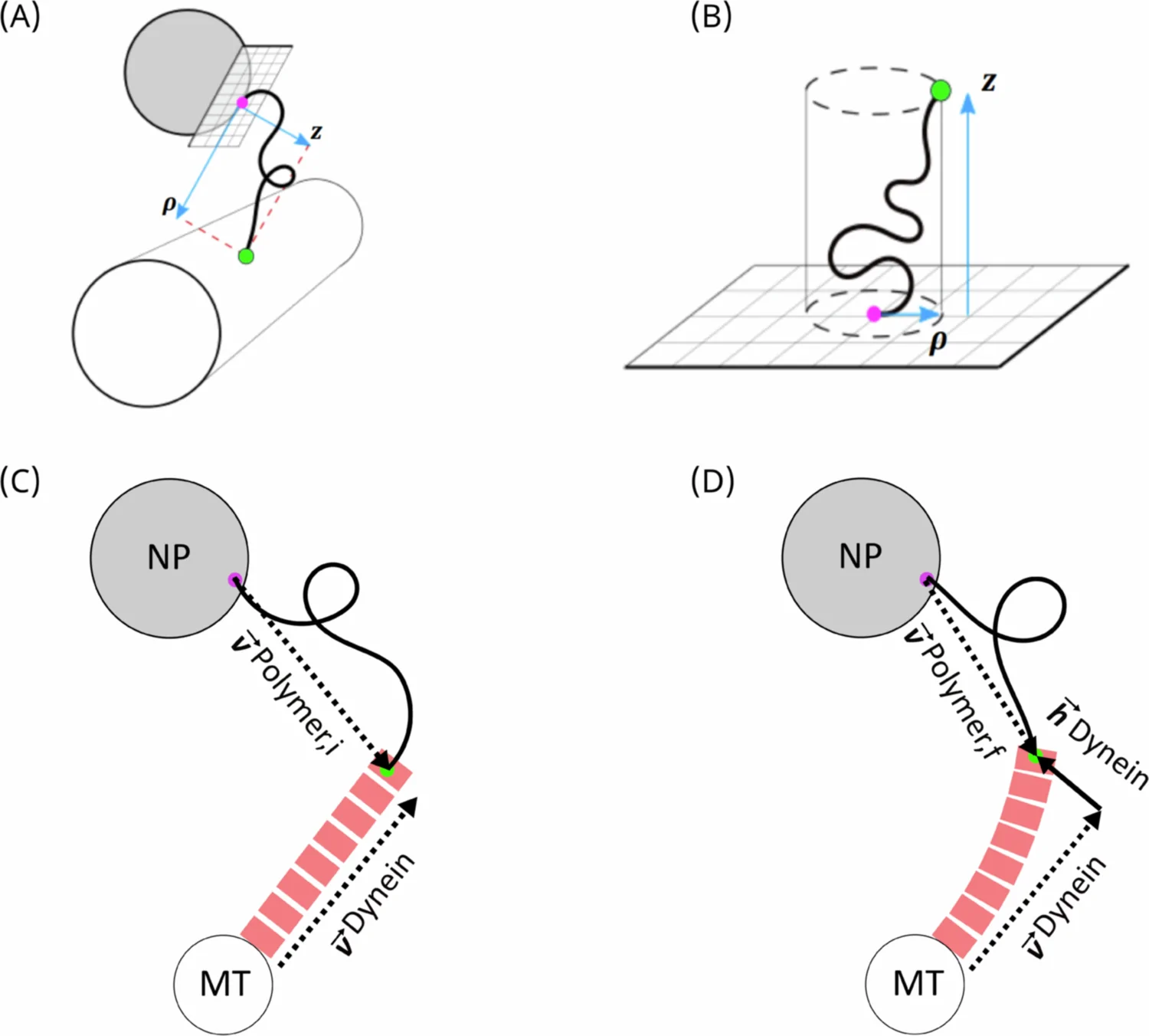
Coupled linker entropy and dynein bending in the energy model. (A) The polymer (black line) connects the nanoparticle surface (pink dot) to the tip of the dynein motor (green dot), shown in cylindrical coordinates relative to a local tangential plane at the anchoring point. (B) Conversion between 3D Cartesian coordinates and cylindrical coordinates used for calculating polymer entropy. (C) The dynein is modelled as a series of connected linear segments in its unbent (rest) configuration (to scale). (D) Upon binding, the dynein bends as each segment deflects by a small angle relative to the one before it ( $\theta_{i}$ ), modelling semi-flexible polymer behaviour. The resulting displacement of the motor’s tip is described by the vector h , which defines the end position of the polymer and governs the energy balance between dynein bending and polymer extension.

#### 2.3.2 Dynein bending

In Ref. Halbi et al. (2023) (Supplementary Material), the dynein was assumed to be a rigid rod standing in the radial direction of the MT cross-section. Here, to better describe the mechanical response of the dynein-importins-PEG complex to force, we account for the dynein semi-flexibility.

Dynein is a semi-flexible polymer that can be described by the continuous worm-like chain (WLC) model on length scales longer than a molecular (short cutoff) scale (Marko and Siggia 1995). To make the model feasible for MC simulations, we use the discrete WLC model (Bustamante et al. 1994) with N rigid segments,

(4) $$\Delta U_{\text{bend}}=\frac{\kappa }{\ell }\sum_{i=1}^{N-1}(1-\cos\theta_{i}),\;\;\;$$

where κ is the bending modulus which determines the persistence length $L_{p}=\kappa /k_{B}T$ , ℓ is the length of the rod-like segments, and $\theta_{i}$ are bending-deflection angles, i.e. the angles between consecutive segments. For the default persistence length, $L_{p}=2\;\mu m$ , we use N=10 , as supported by the numerical convergence analysis presented in Supplemental Section S7 and Figure S5. When $L_{p}$ is varied, N is adjusted according to Equation (S11) in the same section.

The persistence length $L_{p}$ can be estimated by combining results from MD simulations of dynein (Kamiya et al. 2016) with analytical calculations of transverse fluctuations of the end of a semi-flexible rod-like molecule bound to a surface (Appendix A), leading to $L_{p}≃1.8\;\mu m$ . Due to the roughness of this estimate, we use $L_{p}=2\;\mu m$ .

#### 2.3.3 Minimisation

The flowchart of the kinetic MC simulation code is presented in Figure S6. At each candidate attach/detach/step and after each executed event, $\Delta U_{\text{total}}$ is minimised over nanoparticle pose, polymer endpoint (ρ,z) , and dynein bending angles $\left\{\theta_{i}\right\}$ using gradient descent with momentum and line search.

*Optimisation.* Code-level optimisations and parallel execution reduce CPU time by a factor of 100 for full parameter sweeps (e.g. from months to < 3 h on a medium-power workstation), which makes the code practical for external users.

### 2.4 Motor kinetics: attachment, detachment, and stepping

#### 2.4.1 Attachment and detachment (detailed balance)

Let $F_{+}$ and $F_{-}$ be the minimised energies of the connected and disconnected microstates of a particular motor; define $\Delta U_{\mathrm{NP}}=F_{-}-F_{+}$ . Transition rates are (Howard 2001; Klumpp and Lipowsky 2005)

(5) $$k^{-}=\tau^{-1}\frac{1}{1+\exp\;(-\frac{\Delta U_{\mathrm{NP}}}{k_{B}T})},k^{+}=\tau^{-1}\frac{\exp\;(-\frac{\Delta U_{\mathrm{NP}}}{k_{B}T})}{1+\exp\;(-\frac{\Delta U_{\mathrm{NP}}}{k_{B}T})},$$

with τ calibrated such that the single-motor detachment rate at reference conditions equals $k_{0}=1s^{-1}$ (Mallik et al. 2004).

#### 2.4.2 Stepping proposals and acceptance

A bound motor attempts a step $\vec{L}=\left(L_{x},L_{y}\right)$ with probability (Fayer and Granek, 2019; Halbi et al., 2023)

(6) $$P\left(\vec{L}\right)=A\;W\left(\vec{L}\right)\;G\left(\vec{L}\right),$$

where $W\left(\vec{L}\right)$ encodes the intrinsic walking profile (symmetric about the longitudinal axis and completed for missing $\left(\vec{L},-\vec{L}\right)$ pairs) (Reck-Peterson et al. 2006; Gennerich et al. 2007; Qiu et al. 2012), and

(7) $$G\left(\vec{L}\right)=\frac{1}{1+\exp\;\left(-\frac{L_{x}\left(F_{s}-F_{\mathrm{ext}}\right)-U\left(\vec{L}\right)}{k_{B}T_{\mathrm{eff}}}\right)}.$$

Here $F_{s}$ is the stall force, $F_{\mathrm{ext}}$ any external load (zero in our assays), $U\left(\vec{L}\right)$ the energy change for the candidate step after relaxation, and $T_{\mathrm{eff}}=1900K$ is tuned to reproduce the observed zero-load forward/backward ratio (calibration in Supplemental S3) (Gennerich et al. 2007; Elshenawy et al. 2019). This effective temperature is therefore a calibration parameter of the stepping acceptance rule, not an independently measured thermodynamic temperature. Specifically, it was chosen from the zero-load forward/backward step ratio (23% backward and 77% forward); the resulting value, $T_{\mathrm{eff}}=1900$ K, was then held fixed when comparing the model with the non-zero-load walking-distance data summarised in Table S2. The total stepping attempt rate is approximated as the number of bound motors divided by the average stepping interval τ=0.01s (based on $\langle L_{x}\rangle =8\;\mathrm{nm}$ , $\langle V_{x}\rangle =800\;\mathrm{nm}s^{-1}$ ) (Reck-Peterson et al. 2006; Gennerich et al. 2007). Candidate steps must pass the path-wise steric check; otherwise they are recorded as jamming events.

#### 2.4.3 Assumptions

(i) Yeast and mammalian MTBDs bind the same lattice sites (Redwine et al. 2012); (ii) essential cofactors (e.g. BICD2, dynactin) are present and not rate-limiting (McKenney et al. 2014); (iii) $W\left(\vec{L}\right)$ is constructed from quasi-equilibrium stepping data (Elshenawy et al. 2019); (iv) transverse components $L_{y}$ are inferred from longitudinal statistics consistent with dynein measurements (Qiu et al. 2012); (v) symmetry completion is applied to experimental step datasets; (vi) local detailed balance holds between all elementary transitions under the quasi-equilibrium relaxation (Figure 3): for any microstates X⇄Y ,

$$\frac{k_{X\to Y}}{k_{Y\to X}}=\exp\;\left[-\frac{F\left(Y\right)-F\left(X\right)}{k_{B}T_{⋆}}\right],$$

with F(⋅) the minimised free energy and $T_{⋆}\in \left\{T,T_{\mathrm{eff}}\right\}$ chosen per process (attachment/detachment vs. stepping; see Section 2.4). This enforces reversibility with Boltzmann stationary weights.

**Figure 3.**
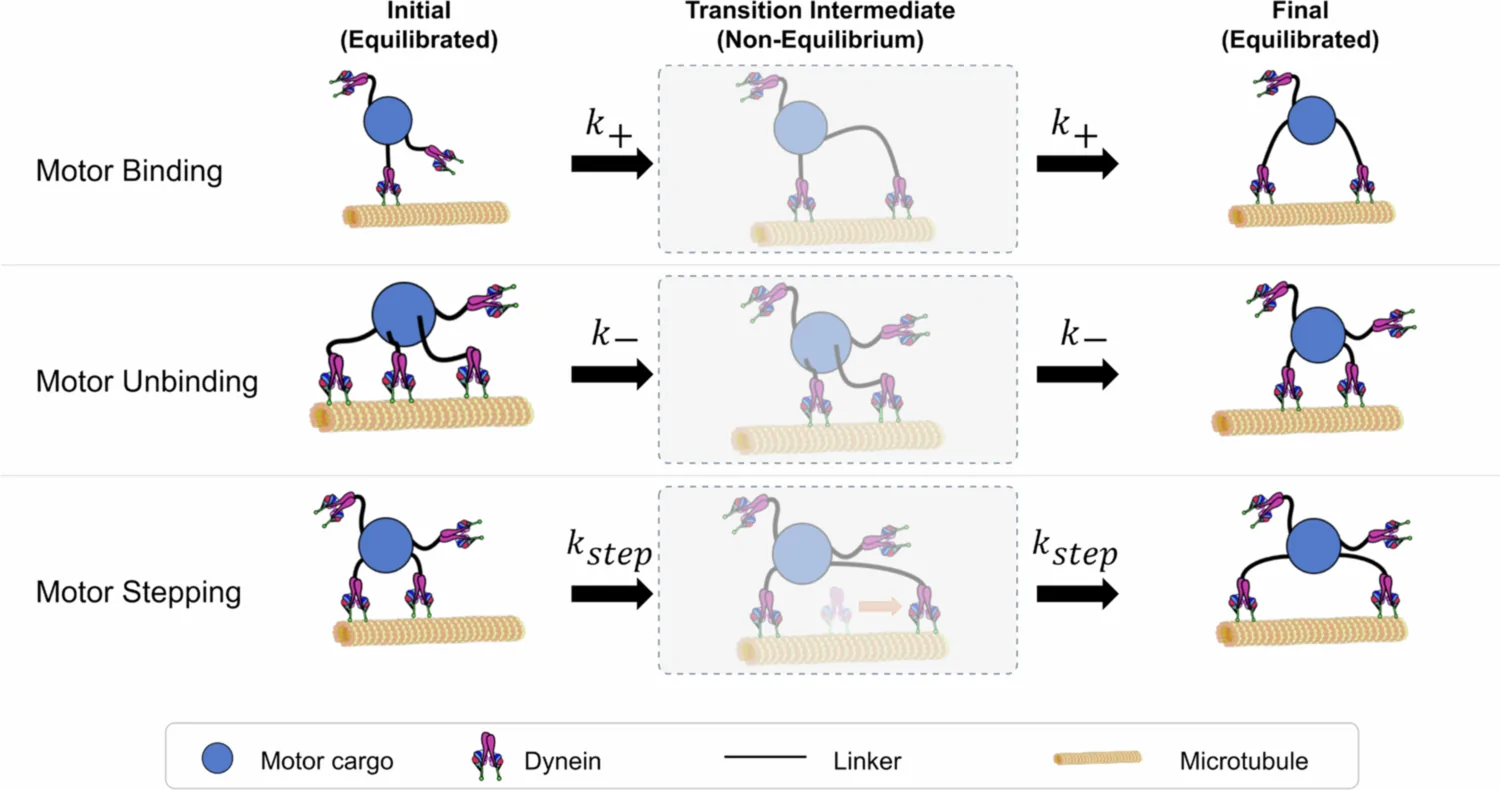
Stochastic motor events and subsequent mechanical relaxation. Schematic representation of the three stochastic events included in the SSA: motor binding (top), motor unbinding (middle), and motor stepping along the microtubule (bottom). Each event begins from a mechanically equilibrated configuration (left). The stochastic event, occurring with the corresponding rate $k_{+}$ , $k_{-}$ , or $k_{\mathrm{step}}$ , instantaneously changes the motor-microtubule configuration and produces a transient non-equilibrium intermediate (centre). The system is then mechanically relaxed by energy minimisation to obtain the new equilibrated configuration (right), allowing the nanoparticle position and orientation and the dynein-linker conformations to adjust. This relaxed configuration serves as the initial state for the subsequent stochastic event. Schematic; not to scale

### 2.5 Model assumptions and quasi-equilibrium justification

We assume a separation between fast mechanical relaxation and slower binding/unbinding/stepping events.

#### 2.5.1 Polymer relaxation

With Zimm scaling, $\tau_{z}=0.325\;\eta R_{0}^{3}/\left(k_{B}T\right)$ , we obtain $\tau_{z}\approx 12\;\mathrm{ns}$ for $R_{0}=5.6\;\mathrm{nm}$ (Supplemental S1), much shorter than stochastic simulation algorithm (SSA) waiting times (Zimm 1956). This implies that the polymer samples all its configurational phase space, which justifies the use of a polymer entropy and elastic free energy.

#### 2.5.2 NP translational and rotational relaxation

Using $K_{p}=3k_{B}T/R_{0}^{2}$ and Stokes drag $\gamma_{t}=6\pi \eta R$ , the translational relaxation time is $\tau_{\mathrm{trans}}=\gamma_{t}/K_{p}\approx 0.85µs$ . The rotational analogue $\tau_{\mathrm{rot}}=8\pi \eta RR_{0}^{2}/\left(3k_{B}T\right)$ is of the same order (Happel and Brenner 1983; Doi and Edwards 1986; Dhont 1996). Both are negligible on SSA timescales, justifying instantaneous relaxation; linear response supports identical timescales for near-equilibrium fluctuations (Kubo 1966).

### 2.6 Experimental

Multi-dynein-coated NPs were prepared as detailed in Refs. Halbi et al. (2021, 2023). In short, fluorescent carboxylated polystyrene NPs were prepared by sequential surface functionalization to enable controlled attachment of NLS peptides and subsequent recruitment of the mammalian nuclear import and dynein transport machinery. Bare NP were first incubated with NeutrAvidin, allowing electrostatic and affinity-based adsorption onto the NP surface. Following removal of excess protein by dialysis, remaining exposed regions were passivated by incubation with BSA, and unbound material was eliminated by centrifugation. The particles were then incubated with biotin–PEG–thiol (5 kDa), providing a defined and saturating PEG layer; excess PEG was removed by repeated centrifugation. NLS peptides were covalently coupled via a bromine–thiol reaction with the PEG terminal group, yielding NPs presenting a controlled surface density of PEG-NLS conjugates. After removal of excess peptides, the functionalized NPs were incubated with HeLa cytoplasmic extracts, promoting the recruitment of α - and β -importins, dynactin, and cytoplasmic dynein motors to the NP surface. The NPs were washed, resuspended in BRB80 supplemented with Mg-ATP, and kept on ice for up to 3 h to preserve motor activity. Flow chambers for motility assays were assembled from cleaned glass slides and coverslips separated by Parafilm spacers, then functionalized by sequential incubation with biotinylated casein and NeutrAvidin to generate a biotin-binding surface compatible with NP immobilisation and motor-driven motility measurements. For motility assays, taxol-stabilised microtubules (40 nM) were introduced into the flow chamber, incubated for 10 min at 23 °C, and washed with three volumes of WshB. Nanoparticles were pelleted (6800 × g, 8 min, 4 °C), resuspended in motility buffer (BRB80 with paclitaxel, glucose, glucose oxidase, catalase, DTT, methyl cellulose, and 10 mM Mg-ATP), and incubated on ice for 5 min before being equilibrated to 23 °C. Motility assays were performed using bare 20 nm radius NP (Halbi et al. 2021, 2023); the results are summarised in Table S1.

## 3 Results and discussion

To analyse the effect of dynein semi-flexibility, we perform two parallel sets of simulations: (i) one in which the dynein behaves as a rigid rod (similar to the model described in Refs. Halbi et al. (2021)), and (ii) the other in which the dynein is semi-flexible, as described in Section 2.3.2. We emphasise, however, that even for the rigid rod case, the present simulations include other features, such as the path-wise excluded volume and improved mechanical energy minimisation. Therefore, the comparison is focused on the semi-flexibility property of dynein.

All the NPs simulated here have a radius of R=20 nm as a typical size of cargo used in drug delivery applications, and we vary two other important parameters: the mean number of NP-bound dynein motor proteins ( $\bar{n}$ ), and the contour length of the PEG spacer polymer ( $L_{c}$ ) (which is proportional to its molecular weight). For each type of these NP parameters, we simulated 1000 NP individual trajectories, over which the relevant observables are averaged. Each trajectory is a separate stochastic realisation of the kinetic Monte Carlo process, with motor grafting geometry and stochastic event sequence independently sampled for that realisation; trajectories are therefore treated as independent replicates for estimation of the mean. Below, we compare the results from two models of dynein rigidity, and compare these to the motility assay results.

The PEG contour length $L_{c}$ is varied here as a design variable: for a PEG chain the contour length is set by its molecular weight and the repeat-unit molecular size. It is therefore intentionally scanned to determine how motility depends on linker length and to locate the maxima in run distance and processivity time. By contrast, the PEG Kuhn length a is a molecular parameter independently constrained by experiment and molecular simulation; reported values for PEG in water at room temperature cluster near 0.7 – 0.76 nm, supporting the value used here (Lee et al. 2008). We therefore do not treat a as a free calibration parameter in the sensitivity analysis. Likewise, the conditional binomial motor-number distribution in Supplemental Section S8 follows from the stated independent-site adsorption model and the restriction to particles carrying at least one motor; its role is to represent the experimentally relevant distribution of motor loadings rather than to introduce another adjustable model form.

### 3.1 Nanoparticle motility versus motor number

Our results are summarised in Figure 4 and Supplementary Table S3. Both semi-flexible and rigid dynein show suppression of longitudinal velocity $V_{x}$ with increasing $\bar{n}$ , with the semi-flexible system showing a weaker velocity suppression. For all three experimental benchmarks ( $\bar{n}\approx 1,2,6$ ), the decrease of the semi-flexible model velocity closely matches that of the measured longitudinal velocity (within 95% CIs), whereas the rigid-motor variant overestimates the reduction (Figure 4A). This reduction of the velocity in the semi-flexible case is also weaker than that which appeared in the previous model (Halbi et al. 2021), despite the addition here of path-wise steric exclusion that censors non-physical trajectories blocked by intervening motors (Vilfan 2005; Leduc et al. 2012). As n increases, this steric interference elevates the fraction of rejected hopping attempts (‘jamming’). However, motor bending compliance, which redistributes deformation between dynein and spacer polymer, permits energetically favourable steps that are disallowed for a rigid motor, which makes a stronger effect (Driver et al. 2017).

**Figure 4.**
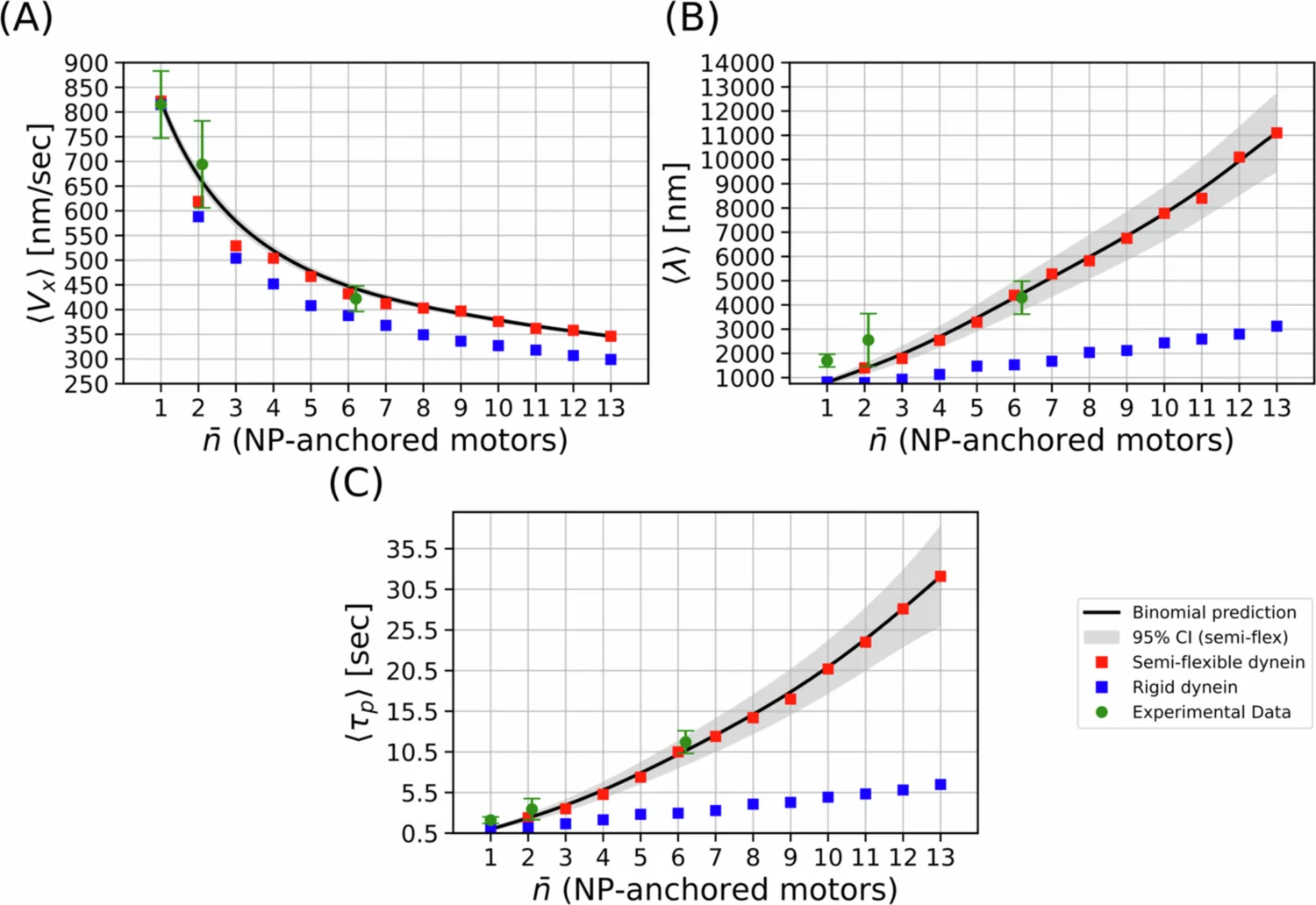
Motility properties against mean motor number **$\bar{n}$ **. Simulation results for the semi-flexible dynein model (red symbols) and the rigid dynein model (blue symbols)—both accounting for path-wise steric exclusion and performed at deterministic numbers ( n ) of NP-attached motors. The black line represents the semi-flexible dynein model accounting for a binomial distribution for n (whose mean is $\bar{n}$ )—see Supplemental S6—which is required for comparison with experimental data (green symbols) for $\bar{n}≃6$ , taken from Ref. Halbi et al. (2023). (A) Mean longitudinal velocity $V_{x}$ decays with $\bar{n}$ ; the semi-flexible model closely matches experiment, whereas the rigid dynein model overestimates the decay. (B) Mean run distance λ and (C) mean run time $\tau_{p}$ increase strongly with $\bar{n}$ ; semi-flexible predictions fall within experimental 95% CIs for the tested conditions at n=2,6 . Shaded bands show the binomially weighted mean ±1.96SEM , as described in Supplemental Section S8. The rigid dynein predictions are far from the experimental data, with a significantly slower increasing gradient.

Regarding the NP processivity characteristics, for $\bar{n}\approx 2$ and $\bar{n}\approx 6$ , the semi-flexible model run-length λ and run time $\tau_{p}$ fall within the experimental 95% confidence intervals; for $\bar{n}\approx 1$ a small systematic deviation persists (Figure 4B,C) (Halbi et al. 2021, 2023). The rigid motor model, on the contrary, shows a much weaker increase in both λ and $\tau_{p}$ , whose values are far from the experimental data.

Mechanistically, the stronger agreement of the semi-flexible model follows from geometry and energetics: a 60nm motor attached to a 20nm NP strongly perturbs the spacer polymer length as a function of azimuthal binding angle; allowing dynein bending relaxes this constraint and increases the set of accessible low-energy configurations, thereby supporting longer run-times and run lengths at higher n (Vilfan 2005).

### 3.2 Effect of polymer contour length on motility

We next vary the PEG $L_{c}$ to identify drug-delivery-relevant design optima associated with maximal run-length. This is so, since during intracellular transport, the longer the run-length for the active motion on one of the MTs, the less diffusion the NP will have to undertake before mounting on the next NP.

We focus here on a system with $\bar{n}=6$ motors anchored on the NP surface, for clarity. For both rigid and semi-flexible dynein, $V_{x}$ increases with increasing $L_{c}$ (Figure 5A and Supplemental Table S4). In the rigid case, a shallow dip appears at very short linkers. This is likely due to additional motors that can bind but are constrained to smaller steps caused by the high energy barrier when attempting to stretch a short polymer.^1^ At vanishing linker length, the rigid model shows a high initial velocity because effectively only a single MT-facing motor can attach and propel the cargo. As the spacer polymer length rises, the velocity reduces (up to the dip) due to the additional binding of motors to the MT, which causes obstruction of their collective motion. Velocities then increase monotonically since the increased flexibility reduces the elastic coupling between motors, which tends to inhibit the motion (Redwine et al. 2012).

**Figure 5.**
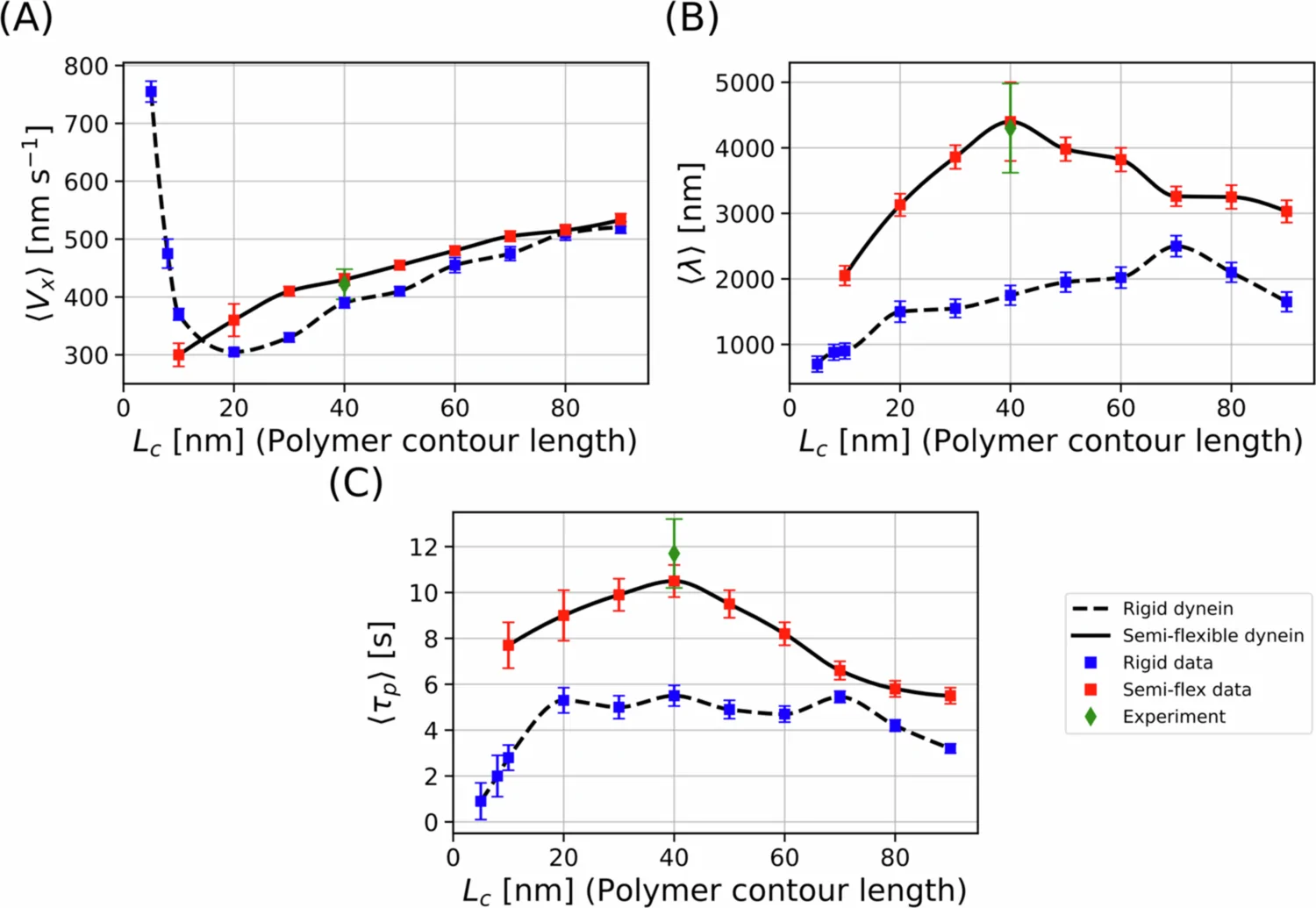
Polymer-length dependence of motility. Simulation results for a system with six motors anchored on the NP surface ( n=6 ); experimental data (green symbols) for $L_{c}≃40$ nm are taken from Ref. Halbi et al. (2023). (A) Mean longitudinal velocity $V_{x}$ versus PEG $L_{c}$ shows a moderate increase for both rigid and semi-flexible dynein, with a short-linker dip only in the rigid case. (B) Mean run distance λ and (C) mean run time $\tau_{p}$ display clear optima and subsequent decline with further lengthening. The semi-flexible model yields an optimum near ~ 40nm for 20nm radius cargos; the rigid model shifts the optimum to longer linkers. Error bars show the mean ±1.96SEM across approximately 1000 runs per condition, as described in Supplemental Section S8.

Turning to the processivity variables, both models exhibit a non-monotonic dependence on $L_{c}$ (Figure 5B,C and Supplemental Table S4): λ and $\tau_{p}$ rise to a maximum before declining. Importantly for design, the semi-flexible model predicts an optimum near ∼40nm for both λ and $\tau_{p}$ . Here $L_{c}$ is deliberately scanned as a design variable, not as an uncertainty parameter: changing $L_{c}$ corresponds to changing PEG molecular weight, and the purpose of the scan is to map the motility landscape and identify the linker length that maximises run length and/or processivity time. This value is obtained for 20nm cargo radius, yet we expect a similar maximum for other NP radii. Interestingly (and partially accidentally), this length of 40nm is just about that used in the reported experiment whose data is shown on the plots (Halbi et al. 2021, 2023). For comparison, the rigid dynein model shifts the optimum to longer spacer polymers, likely due to missing bending degrees of freedom (Bustamante et al. 1994). Past the optimum, further spacer polymer lengthening increases entropic penalties for anchors near the NP–MT contact region, while having a weaker effect on the velocity, which reduces the number of MT-bound motors and is most likely the reason behind the reduction in processivity (Cohen and Granek 2014).

### 3.3 Additional motility observables

We report three observables that, while challenging to quantify experimentally, illuminate model mechanisms and guide design (Figure 6): (i) active motors, (ii) mean angular velocity, and (iii) helical pitch.

(i) By “active motors” we refer to simultaneously bound motors during a single NP run, whose mean number is denoted by $M_{b}$ , see Figure 6A. In the rigid-motor model, the number of active motors saturates near $M_{b}∼2.2$ with increasing $\bar{n}$ , due to large (even if less frequent) steps—whose frequency is increased due to crowding—that cause highly tensed PEGs, which in turn enhance the unbinding rate. Conversely, the semi-flexible model shows a monotonic increase without an apparent saturation over the explored range. This may be associated with a relatively reduced tension due to dynein bending (for the longer steps), resulting in a more moderate increase in the unbinding rate.
(ii) The mean angular (azimuthal) velocity 〈ω〉 decays strongly with $\bar{n}$ in both models (Figure 6B), more moderately for semi-flexible dynein (i.e. it is always larger for the semi-flexible case), reflecting increased jamming and partial angular force cancellation as more motors engage. The fluctuations in the mean angular velocity, reflected by the standard deviation, are much higher for semi-flexible dynein. Moreover, while the angular velocity standard deviation of semi-flexible dynein increases dramatically with increasing $\bar{n}$ , that of rigid dynein is slowly decreasing. This behaviour reflects the enhanced number of transverse MT-bound motors in the semi-flexible case, relative to the rigid case, leading to large angular fluctuations.
(iii) Lastly, we quantify the mean kinematic pitch, $\langle H_{\mathrm{kin}}\rangle$ , which follows directly from time-averaged velocities:

**Figure 6.**
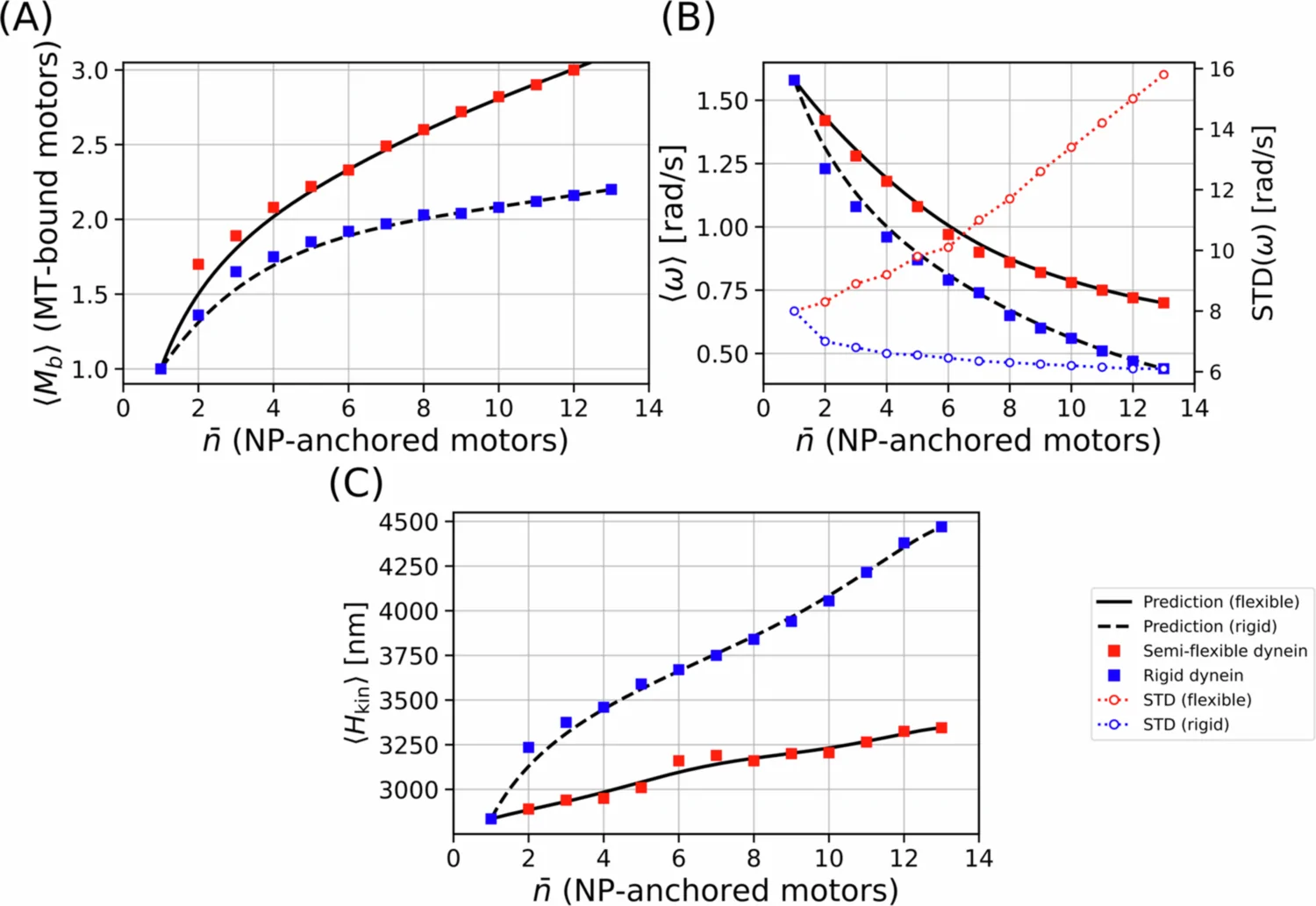
Additional motility observables versus motor number. (A) Mean number of simultaneously active motors ( $M_{b}$ ) increases with $\bar{n}$ ; rigid-dynein effectively plateau, whereas semi-flexible dynein shows continued growth. (B) Mean angular (helical) velocity about the MT axis decays with $\bar{n}$ ; decay is steeper for rigid motors. (C) Mean kinematic pitch $H_{\mathrm{kin}}=2\pi V_{x}/\langle \omega \rangle$ grows markedly with $\bar{n}$ in the rigid-dynein case but remains nearly constant for semi-flexible dynein, consistent with reduced backtracking and more regular trajectories when bending compliance is present.

(8) $$\langle H_{\mathrm{kin}}\rangle =\frac{2\pi \langle V_{x}\rangle }{\langle \omega \rangle },$$

where $\langle V_{x}\rangle$ is the mean longitudinal velocity and 〈ω〉 the mean angular velocity, Figure 6C. $\langle H_{\mathrm{kin}}\rangle$ for semi-flexible dynein is always smaller than for the rigid case and increases slowly as $\bar{n}$ grows, while the rigid-motor system exhibits a pronounced rise. These dependencies reflect mainly the dependencies of 〈ω〉 on $\bar{n}$ (with some weaker influence of $V_{x}$ ).

## 4 Discussion and conclusions

We introduced a three-dimensional, energy-based simulation of multi-motor nanoparticle transport that treats dynein as a semi-flexible polymer and enforces path-wise steric exclusion along candidate stepping trajectories. Using standardised biophysical parameters, the model quantitatively reproduces previously published measurements with mounting dynein loading—velocity suppression, and strong enhancement of run distances and run times—closing key gaps observed in earlier rigid-motor, destination-only exclusion frameworks (Halbi et al. 2021, 2023).

Quantitative comparisons with experimental data are available for a 20 nm radius NP with three values of mean bound motors: $\bar{n}\approx 1$ , $\bar{n}\approx 2$ , and $\bar{n}\approx 6$ . Experimentally, NPs were prepared with different PEG-NLS surface densities and different dilutions of HeLa cell cytoplasmic extracts, and the value of $\bar{n}$ was deduced using a Western Blot assay; details can be found in Section 2.6 and Refs. Halbi et al. (2021, 2023). Remarkably, model predictions for $\bar{n}\approx 2$ and $\bar{n}\approx 6$ lie within the experimental 95% confidence intervals and close the gap between theory and experiment. Beyond these benchmarks, our simulations provide predictions over the full $\bar{n}=1-13$ range. The remaining deviation at $\bar{n}\approx 1$ is small but systematic. We cannot attribute it uniquely to a single microscopic effect; it may reflect conformational and kinetic features of an isolated dynein–importins complex that are not resolved by the coarse-grained WLC representation and the common parameter set used for multi-motor transport. A local one-at-a-time sensitivity analysis shows that the qualitative motor-number dependence of the predicted motility observables is preserved across the tested parameter ranges (Supplemental Section S4).

A central outcome is a set of actionable design rules for dynein-powered, nucleus-directed delivery. For ∼20 nm-radius cargos hauled by six anchored motors, scanning the PEG $L_{c}$ (i.e. its molecular weight) reveals a clear, non-monotonic processivity landscape with a maximum near ∼40 nm (PEG molecular weight of 5 kDa). The longest achieved run-length is around 4–5μm , suggesting a five-fold increase in active transport distance compared to a single-dynein-transported cargo. Increasing the number of engaged motors reliably lengthens run-distance and run-time, while reducing the longitudinal velocity, thus providing a tunable trade-off between speed and persistence.

These results follow mechanistically from two ingredients essential to cooperative transport: bending compliance, which expands low-energy binding/stepping configurations, and path-wise steric exclusion, which correctly penalises trajectory conflicts as motor number grows. Our cargo transport model and computer code allow us to repeat this optimisation procedure for different NP sizes and bound motor numbers.

The present work has several limitations. Quantitative validation relies chiefly on the available motility measurements for 20-nm-radius polystyrene NPs in HeLa cytoplasmic extracts (Halbi et al. 2021, 2023); thus, the model is validated at the level of microtubule-based transport rather than at the level of complete nuclear delivery. In particular, the calculated optimum in $L_{c}$ should be interpreted as an optimum for microtubule transport processivity within the present model, not as a prediction of maximal nuclear uptake. Nuclear delivery additionally depends on endocytosis, endosomal escape, cytoplasmic crowding, microtubule organisation and microtubule-associated proteins (MAPs), competing motors, and nuclear-pore transport, none of which are represented explicitly here. In a more crowded cytoplasm, steric obstacles could alter the balance between linker extension, motor accessibility, and cargo detachment; whether this shifts the optimum toward shorter or longer PEG therefore requires an explicit extension of the present model rather than an a priori conclusion. By contrast, the *microscopic* cytoplasmic viscosity needs to be higher than the water viscosity by several orders of magnitude (associated with a proportional increase of the hydrodynamic drag) to fail the assumption of ‘infinitely fast’ mechanical equilibration of the NP position and orientation (as compared to motor stepping and binding/unbinding processes, see Section 2.4), a very uncommon situation. MAP-mediated obstacles could be incorporated as additional spatially heterogeneous forbidden regions or modified transition rates along the MT lattice. The microtubule lattice is otherwise treated as homogeneous and static, and a modest single-motor discrepancy remains. Future studies should pair expanded simulations with new measurements spanning linker lengths and NP sizes, incorporate heterogeneous binding landscapes and cytoplasmic crowding, and probe additional readouts (e.g. active-motor counts, angular velocity, and pitch) to further stress-test the mechanism. More practically, as the model assumes a given number of NP-attached motors $\bar{n}$ as an input, this number has to be calibrated (using similar Western Blot assays (Halbi et al. 2023)) for any type of cells (other than HeLa) and for other PEG-NLS surface densities and NP sizes. Our model can also be modified to describe ‘lipid nano-particles’ (i.e. lipid vesicles) (Mehta et al. 2023), which will incorporate a certain amount of PEG-lipids, each ending with a NLS (i.e. lipid-PEG-NLS complexes). Here, the fluidity of the membrane and membrane flexibility would need to be addressed explicitly.

With these extensions, the framework presented here can serve both as a predictive engine for optimising multi-motor, nucleus-directed NP delivery and as a tool for dissecting the biophysical principles that govern cooperative, dynein-based transport while substantially lowering experimental burden through fast, in silico screening. For example, potentially, our model opens a route to study (theoretically) the intracellular dynein-mediated transport of viruses, such as HIV (Dharan and Campbell 2018; Carnes and Aiken 2019), Herpes Simplex (Döhner et al. 2002), and various adenoviruses (Kelkar et al. 2004; Scherer and Vallee 2011), as these viruses express dynein recruiting ligands (NLS peptides for HIV and Herpes Simplex, hexon proteins in case of adenoviruses). Our new assessment of the important role of the dynein semi-flexibility may suggest their recruitment of a few motors and explain their relatively high motility and peri-nuclear localisation (Döhner et al. 2002; Kelkar et al. 2004; Dharan and Campbell 2018; Halbi et al. 2023).

As discussed above, optimising the run-length and/or run-time of cargo transport over microtubules is not necessarily identical to optimising the final nuclear cargo uptake, as this involves other crucial processes, such as endocytosis, endosomal escape, and passage through the nuclear pore. Interestingly, however, at least for the 20 nm NP with 40 nm PEG $L_{c}$ (i.e. 5 kDa molecular weight), it was recently shown that these other processes are quite efficient, even if not optimised with certainty (Halbi et al. 2023). This is partly because NLS also serves as a reasonable cell-penetrating peptide, and suggests that optimal nuclear uptake may be achieved with fine-tuning of the NP characteristics.

## Supplementary Material

Supplemental_Revised_MARKED_FOR_SUBMISSION.pdf Supplemental_Revised_MARKED_FOR_SUBMISSION.pdf

## Data Availability

The authors confirm that the data supporting the findings of this study are available within the article and its supplementary materials. The simulation code used to generate the results presented in this study is publicly available on GitHub at https://github.com/tyagev-dotcom/dynein-transport-simulation.

## Endnote

^1^
We expect such a minimum also for the semi-flexible case, though at shorter spacer polymer $L_{c}$ , yet the simulations did not provide a converging velocity value; therefore, the data is not shown for these short polymer lengths.

## Appendix A. Persistence-length estimate for dynein MTBD

In Ref. Kamiya et al. (2016), MD simulations of dynein in which the transverse fluctuations of dynein MTBD are followed, and the authors deduce their variance $\langle h^{2}\rangle$ and the corresponding effective stiffness $k_{b}=k_{B}T/\langle h^{2}\rangle$ . Here we translate these values into a persistence length parameter $L_{p}$ that is used in the WLC model (Granek 1997; Broedersz and MacKintosh 2014).

Consider a semi-flexible rod-like polymer that is bound at one of its ends (set to x=0 ), and free at the other ( x=L ). We describe the displacements from straightness by the field h(x) , which is subject to the boundary conditions: h(0)=0 and ${\partial h/\partial x|}_{0}=0$ . Expanding h(x) in a sine Fourier series (thus implementing h(0)=0 ), i.e. $h\left(x\right)=\sum_{k}h_{k}\;\sin kx$ , the wavenumbers k are found to be quantised (by implementing ${\partial h/\partial x|}_{0}=0$ ) as $k_{n}=\left(n+1/2\right)\pi /L$ , n=0,1,2,… . The WLC free-energy and equi-partition theorem implies $\langle h_{k}^{2}\rangle =2k_{B}T/\left(\kappa k^{4}\right)$ with $\kappa =k_{B}TL_{p}$ , leading to the following variance of fluctuations at x=L ,

(A1)
$$\langle h^{2}\left(L\right)\rangle =\frac{2L^{3}}{L_{p}}\sum_{n=0,1,\ldots }{\left(\pi /2+n\pi \right)}^{-4}=\frac{L^{3}}{3L_{p}}$$

in which the numerical prefactor, 1/3 , differs from the one obtained for a rod-like polymer that is free at both ends (Broedersz and MacKintosh 2014). This translates into the following relation between the stiffness parameter, $k_{b}$ used in Ref. Kamiya et al. (2016) and the persistence length $L_{p}$

(A2)
$$L_{p}=\frac{k_{b}L^{3}}{3k_{B}T}$$

If we use L≃13 nm for the dynein length, and the typical value obtained in the simulations ‘ADP model’ (Kamiya et al. 2016), i.e. when ADP is bound to the motor rather than ATP, $k_{b}≃10$ pN/nm, which is the one appropriate for a non-stepping dynein (see Table II in Kamiya et al. (2016)), we obtain an estimate for persistence length parameter value $L_{p}≃1.8\mu$ m. Considering the possible errors in this estimate (since it is based solely on MD simulations and theory), we conclude that it is more than sufficient to take in our MC simulations $L_{p}≃2\mu$ m.
