# Supplementary material for: Modelling nano-particle-PEG-NLS complexes for nucleus-targeted drug delivery: revisiting the multi-dynein nano-cargo transport on microtubules
Source: Drug Deliv. 2026 Oct 12;33(1):2738276. doi: 10.1080/10717544.2026.2738276 (PMC13665706; doi:10.1080/10717544.2026.2738276)
Supplement: Supplementary file 1 [file IDRD_A_2738276_SM7925.pdf]

Supplemental Material

Modeling Nano-Particle-PEG-NLS Complexes for Nucleus-Targeted Drug Delivery: Revisiting the Multi-Dynein Nano-Cargo Transport on Microtubules

Tal Yagev[1], Itay Fayer[1], Itay Adar[2], Gal Halbi[2,#], Anne Bernheim-Groswasser[2,3], Rony Granek[1,3,4,*]

[1]Avram and Stella Goldstein-Goren Department of Biotechnology Engineering, Ben-Gurion University of the Negev, Beer Sheva 84105, Israel
[2]Department of Chemical Engineering, Ben-Gurion University of the Negev, Beer Sheva 84105, Israel
[3]Ilse Katz Institute for Nanoscale Science and Technology, Ben-Gurion University of the Negev, Beer Sheva 84105, Israel
[4]Department of Physics, Ben-Gurion University of the Negev, Beer Sheva 84105, Israel

*Corresponding author. `Email:rgranek@bgu.ac.il`
#Present address: Dept. of Chemistry, NRCN, Beer-Sheva 84190, Israel

S1. Estimation of the PEG Contour Length, L_c, and Radius of Gyration, R_g

In our motility experiments, we use biotin–poly(ethylene glycol)–thiol (Biotin–PEG–thiol) with a nominal molecular weight of 5 kDa. The molar mass of a single ethylene glycol (EG) repeat unit (–CH_2–CH_2–O–) is 44.05 g mol^{-1}. Therefore, the number of EG subunits in a 5 kDa PEG chain is

$$n \;=\; \frac{5000}{44.05} \;\approx\; 114.$$

To estimate the contour length L_c and the radius of gyration R_g, we use the EG subunit length d_{EG} [1] and the PEG Kuhn length $a = 0.76$ nm [2]. Assuming an ideal (Gaussian) chain [2, 3]:

$$L_c \;=\; d_{\mathrm{EG}}\, n, \tag{S1}$$

$$L_c \;=\; N_K\, a \;=\; d_{\mathrm{EG}}\, n, \tag{S2}$$

$$N_K \;=\; \frac{d_{\mathrm{EG}}\, n}{a}, \tag{S3}$$

$$R_0 \;=\; a\,\sqrt{N_K}, \tag{S4}$$

$$R_g \;\simeq\; \frac{R_0}{\sqrt{6}} \;=\; \frac{a}{\sqrt{6}}\sqrt{N_K} \;=\; \sqrt{\frac{a\, d_{\mathrm{EG}}\, n}{6}}. \tag{S5}$$

S1.1. Numerical estimate (5 kDa PEG)

Using $d_{\mathrm{EG}} = 0.358$ nm [1], $n \approx 114$, and $a = 0.76$ nm [2]:

$$L_c \approx 0.358 \times 114 \approx 40.8 \text{ nm}, \qquad N_K \approx \frac{40.8}{0.76} \approx 53.7,$$

$$R_0 \approx 0.76\sqrt{53.7} \approx 5.57 \text{ nm}, \qquad R_g \approx \frac{5.57}{\sqrt{6}} \approx 2.27 \text{ nm}.$$

These values provide the PEG spatial scales used in the simulation (ideal-chain limit). Polydispersity of commercial 5 kDa PEG and solvent quality can shift R_g moderately; the above serves as a consistent baseline with the Gaussian-chain assumption.

S2. Experimental Results

Table S1.: Experimental motility metrics. V_x is the longitudinal velocity; V_y is the transverse velocity; τ_p is the run-time; λ^* is the absolute accumulated distance; λ is the run-length, i.e., the net accumulated distance. In the last two rows, f/b denote forward/backward steps, and r/l denote right-/left-handed steps. Data are taken from Refs. [28]-[29] of the main text.

	**I**	**II**	**III**	**IV**	**V**
[NLS] [μM]	0.025	0.05	0.05	0.3	0.3
$\langle N \rangle$	5.2	10.4	10.4	36.6	36.6
[CE] [mg/mL]	3.4	3.4	3.4	3.4	6.8
Number of steps	80	62	428	134	536
$\langle \lvert V \rvert \rangle$ [nm/s]	905 ± 18	793 ± 16	847 ± 33	493 ± 23	320 ± 20
$\langle V_x \rangle$ [nm/s]	815 ± 68	769 ± 49	619 ± 39	422 ± 26	72 ± 22
$\langle V_x \mid V_x > 0 \rangle$ [nm/s]	939 ± 57	783 ± 48	687 ± 18	476 ± 22	253 ± 20
$\langle V_x \mid V_x < 0 \rangle$ [nm/s]	-305 ± 141	-91 ± 0	-609 ± 160	-224 ± 76	-319 ± 43
$\langle V_y \rangle$ [nm/s]	1.7 ± 25	-0.07 ± 24	206 ± 27	67 ± 16	39 ± 10
$\langle V_y \mid V_y > 0 \rangle$ [nm/s]	190 ± 24	173 ± 23	278 ± 24	92 ± 13	132 ± 9
$\langle V_y \mid V_y < 0 \rangle$ [nm/s]	-152 ± 23	-143 ± 16	-299 ± 62	-277 ± 104	-162 ± 18
$\langle \tau_p \rangle$ [s]	2.1 ± 0.4	3.0 ± 0.8	3.9 ± 0.5	11.7 ± 1.4	23.3 ± 4.1
$\langle \lambda^* \rangle$ [nm]	2200 ± 300	2600 ± 540	3800 ± 720	4900 ± 750	7700 ± 1100
$\langle \lambda \rangle$ [nm]	1700 ± 260	2300 ± 480	2800 ± 610	4300 ± 680	1700 ± 600
Fractions of forward and backward steps	0.9 f | 0.1 b	0.98 f | 0.02 b	0.9 f | 0.1 b	0.92 f | 0.08 b	0.67 f | 0.33 b
Fractions of right- and left-handed steps	0.45 r | 0.55 l	0.45 r | 0.55 l	0.92 r | 0.08 l	0.93 r | 0.07 l	0.67 r | 0.33 l

S3. Effective Temperature Calibration and Model Validation

To achieve a good fit of the parameter T_{eff}, it was previously shown that its value can be adjusted using measured single-motor step statistics (see Ref. [17] of main text for details), so that in the absence of external force ($F = 0$), the backward and forward step fractions match the empirical data: 23% and 77%, respectively, leading to a value of $T_{\text{eff}} = 1900\,\text{K}$. This effective temperature is a calibration parameter of the stepping acceptance rule rather than a thermodynamic temperature; it is fixed by the zero-load forward/backward step ratio and is then kept fixed for the non-zero-load validation summarized below. As suggested in Ref. [17] (of the main text), it may result from active noise associated with partial energy dissipation released by ATP hydrolysis, not used for dynein motor work and associated with finite "engine" efficiency. To further verify these fits, the single-motor model predictions were compared with experimental data for different force values (Ref. [17] of main text). It is worth noting that all experimental data, except those under zero load ($F = 0$), are consistent with the predictions of our model for a single mammalian cytoplasmic dynein, within theoretical and experimental errors.

Table S2.: Comparison of experimental and theoretical walking distances of a single mammalian dynein under different external forces. Experimental uncertainties reflect the FIONA resolution of $\pm 1\,\text{nm}$, combined with the reported standard error of the mean (SEM) as $\sigma_{\text{exp}} = \sqrt{1 + \text{SEM}^2}$. The small discrepancy between model and experiment at $F = 0$ is likely due to experimental error at very low laser trapping powers.

F [pN]	Experimental	Theoretical
0.0	11.4 ± 1.1 nm	9.1 nm
1.5	5.4 ± 1.5 nm	6.9 nm
2.3	4.3 ± 1.5 nm	5.2 nm
2.9	2.5 ± 1.5 nm	3.3 nm
3.6	-0.5 ± 1.5 nm	0.3 nm

S4. Local Parameter Sensitivity Analysis

To assess the robustness of the model predictions, we performed a local one-at-a-time sensitivity analysis around the baseline parameter set. The baseline simulation used a dynein persistence length of $L_p = 2$ μm, a dynein–microtubule binding enthalpy of $8k_BT$, an effective dynein length of 60 nm, and a single-motor detachment rate of 1 s^{-1}. The tested ranges were $L_p = 1$–3 μm, binding enthalpy $= 7$–$9k_BT$, effective dynein length = 54–66 nm, and detachment rate = 0.8–1.2 s^{-1}. Each parameter was varied independently while all others were held at their baseline values. Simulations were performed for 1–13 grafted dynein motors using 250 nanoparticle trajectories per condition.

As shown in Fig. S2, the qualitative motor-number dependence of velocity, run length, and processivity time is preserved across the tested parameter ranges, although the absolute predicted values become increasingly parameter dependent at larger motor numbers. This analysis supports the robustness of the overall trends but does not address "interactions" between simultaneously varied parameters.

The PEG Kuhn length is not included in this sensitivity scan because it is a molecular property independently constrained by experiment and molecular simulations, rather than a freely calibrated transport parameter. Reported values for PEG/PEO in water at room temperature cluster around 0.7–0.76 nm, including 0.76 nm from single-molecule measurements and closely related molecular-simulation estimates [2]. We therefore use $a = 0.76$ nm rather than assigning an arbitrary uncertainty interval to a.

Likewise, the conditional binomial motor-number distribution used in Section S8 follows from the stated independent-site adsorption model and the restriction to the population with at least one attached motor. Its mean is controlled by the motor-loading condition. We therefore do not treat the distributional form itself as a freely adjustable uncertainty parameter.

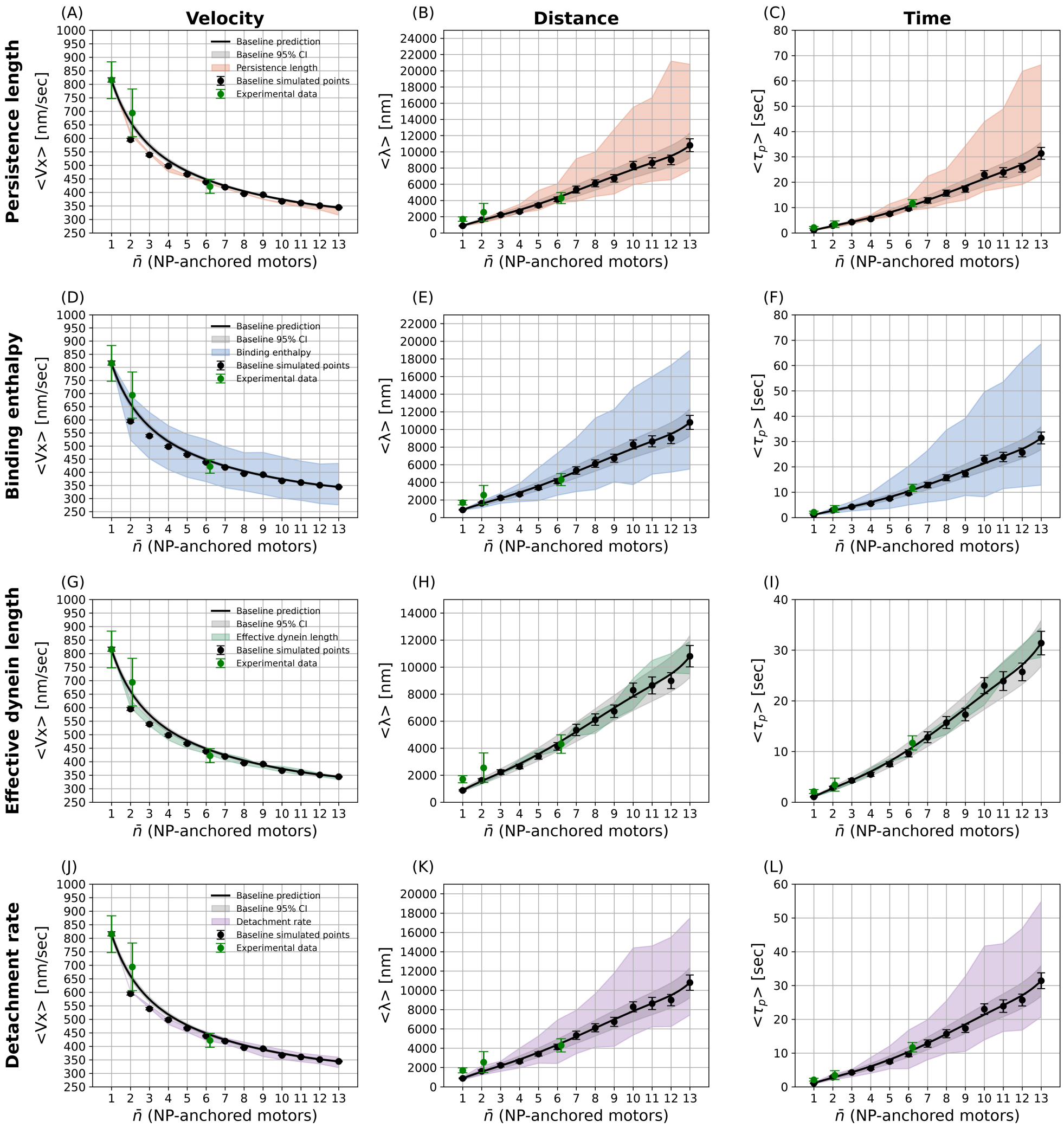

Figure S1.: Separate parameter sensitivity checks for the dynein transport simulation. Each row shows the effect of varying one model parameter around the baseline condition: $L_p = 2\ \mu$m, binding enthalpy $8k_BT$, effective dynein length 60 nm, and detachment rate 1 s^{-1}. The tested ranges were $L_p =$ 1–3 μm, binding enthalpy 7–9k_BT, effective dynein length 54–66 nm, and detachment rate 0.8–1.2 s^{-1}. For each parameter value, 250 nanoparticle trajectories were simulated for each motor number from 1 to 13. Colored bands show the range between the lower and upper tested values for the parameter shown in that row; the gray band shows the 95% confidence interval of the baseline simulation. Columns show mean longitudinal velocity V_x, mean run-length λ, and mean run-time τ_p.

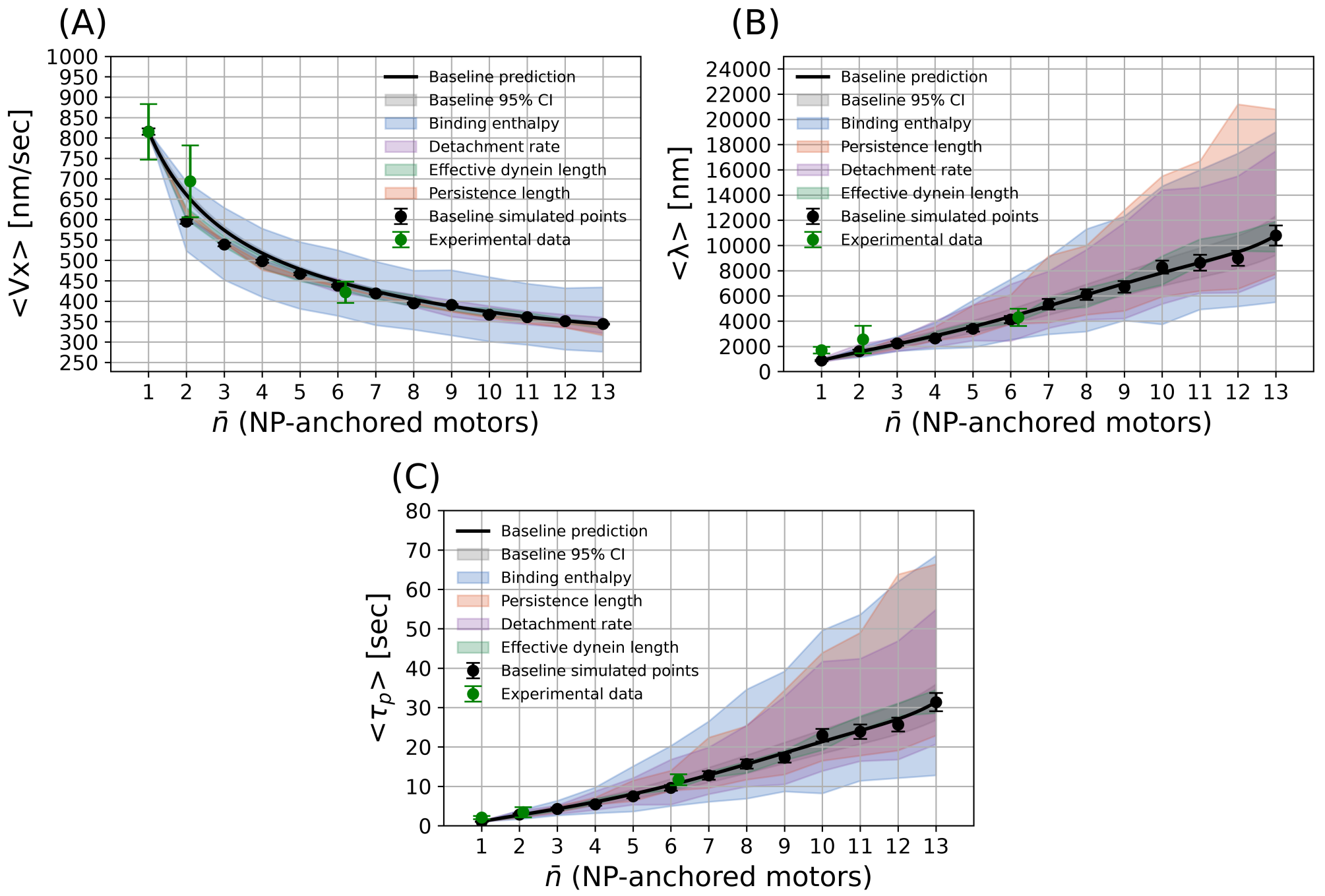

Figure S2.: Combined parameter sensitivity analysis of the dynein transport simulation. Same data as in Fig. S1, presented in a single plot for each of the three observables; see Fig. S1 captions for details. Panels show: (A) mean longitudinal velocity V_x, (B) mean run-length λ, and (C) mean run-time τ_p.

S5. Coordinate Transformation

Since each polymer is anchored at a different point on the nanoparticle (NP) surface, the local tangential plane at its anchor is rotated by a different angle relative to the microtubule-fixed laboratory frame. We therefore perform a coordinate transformation for each polymer to obtain its specific free-energy change ΔU_{motor}.

Let the NP center be the origin. For polymer i, denote the anchor (on the NP surface) and the dynein attachment (free end) by

$$\mathbf{r}_i = (x_i, y_i, z_i), \qquad \mathbf{r}_f = (x_f, y_f, z_f),$$

and define the end-to-end vector

$$\Delta\mathbf{r} \equiv \mathbf{r}_f - \mathbf{r}_i.$$

Let $\hat{\mathbf{n}}_i$ be the outward unit normal at the anchor. For a spherical NP of radius R, $\hat{\mathbf{n}}_i = \mathbf{r}_i/\|\mathbf{r}_i\|$ (with $\|\mathbf{r}_i\| = R$). Decompose $\Delta\mathbf{r}$ into normal and tangential parts as

$$Z \equiv \hat{\mathbf{n}}_i \cdot \Delta\mathbf{r}, \qquad \boldsymbol{\rho} \equiv \Delta\mathbf{r} - Z\,\hat{\mathbf{n}}_i, \quad \rho \equiv \|\boldsymbol{\rho}\|.$$

In this notation, the term $Z^2 + \rho^2$ is precisely the squared end-to-end distance between the anchored and free ends:

$$\|\Delta\mathbf{r}\|^2 = Z^2 + \rho^2 = (x_f - x_i)^2 + (y_f - y_i)^2 + (z_f - z_i)^2. \tag{S6}$$

If φ is the angle between $\Delta\mathbf{r}$ and the anchor normal $\hat{\mathbf{n}}_i$, then $Z = \|\Delta\mathbf{r}\|\cos\varphi$ (and $\rho = \|\Delta\mathbf{r}\|\sin\varphi$). Equivalently, using the scalar product with the unit normal,

$$Z = \hat{\mathbf{n}}_i \cdot \Delta\mathbf{r} = \frac{(x_f - x_i)x_i + (y_f - y_i)y_i + (z_f - z_i)z_i}{\sqrt{x_i^2 + y_i^2 + z_i^2}}. \tag{S7}$$

Equations (S6)–(S7) provide the normal (Z) and in-plane (ρ) components required to evaluate each polymer's contribution to the free-energy change ΔU_{motor} in its local frame without explicitly constructing rotation matrices.

S6. Dynein–Importin Complex Dimensions

Vertical (Height) estimate

For length estimation, the dynein structure (excluding the tail domain) is treated as three serial domains: MTBD ($\sim$4 nm), stalk ($\sim$15 nm), and AAA$^+$ ring ($\sim$10 nm), summing to $\sim$30 nm (PDB entries 3VKH, 3AY1, 6RZA). It is known that roughly two-thirds of the heavy chain (C-terminal) spans from the MTBD to the AAA$^+$ ring, with the remaining third forming the N-terminal "tail" backbone [4]. This mapping yields a total dynein heavy-chain length

$$d_{\text{dyn}} \approx 45 \text{ nm}.$$

The α/β importin complex length is taken as

$$d_{\text{importins}} \approx 15 \text{ nm},$$

recognizing it as a rough estimate between dense and relaxed conformations; the NLS peptide is negligible in size. Thus, the vertical extent from the MTBD to the polymer (PEG) attachment is

$$d_{\text{vertical}} \;=\; d_{\text{dyn}} + d_{\text{importins}} \;\approx\; 60 \text{ nm}.$$

Lateral (Radius) estimate

When a motor attempts a step, there are two steric blocking modes due to the *forbidden volume* of other motors: (i) the target binding site is already occupied, or (ii) another motor lies in the stepping path and occludes it (dynein advances along the lattice rather than via detach–reattach to the target). Upon a blocking event, the motor remains in place (jamming) and the sampled Monte Carlo time increment is added to the total run time.

We define $V_{\text{pair},\langle ij\rangle}$ as the effective volume occupied by a pair of binding sites $\langle ij\rangle$ when including the inter-site space (Fig. S3A). A forbidden-volume blocking event is registered whenever $V_{\text{pair},\langle ij\rangle}$ overlaps $V_{\text{pair},\langle k\ell\rangle}$ with $\langle ij\rangle \neq \langle k\ell\rangle$. Because our coarse-grained model does not resolve the full dynein shape, we estimate the mean center–axis separation that produces blocking, denoted d_{jam}.

To this end, we represent each V_{pair} by its footprint on the local surface—a hemispherical strip of length L (Fig. S3B). Consider two such strips in contact (Fig. S3C–D), forming a mutual angle α (Fig. S4). Let the distances from a reference point to the strip centers be

$$r_1 = \frac{L}{2} - x, \qquad r_2 = \frac{L}{2}, \qquad x \in [0, L],$$

and let u be the center–center distance between the two strip centers. By the law of cosines,

$$u^2(x,\alpha) = r_1^2 + r_2^2 - 2\,r_1 r_2 \cos\alpha = \Big(\tfrac{L}{2} - x\Big)^2 + \Big(\tfrac{L}{2}\Big)^2 - L\Big(\tfrac{L}{2} - x\Big)\cos\alpha. \tag{S8}$$

The configurational average (uniform in x along the strip and in relative angle α over $[0, \pi/2]$) is

$$\langle u\rangle_{\text{config}} = \frac{2}{\pi}\int_0^{\pi/2} d\alpha\, \frac{1}{L}\int_0^L dx\, \sqrt{u^2(x,\alpha)} \;\approx\; 0.543\,L. \tag{S9}$$

Since L varies, we use its mean value,[1] yielding

$$d_{\text{jam}} \;\equiv\; \langle u\rangle_{\text{config}} \;\approx\; 0.543\,\bar{L} \;\approx\; 12 \text{ nm}. \tag{S10}$$

This estimate does not include the "crossing" configuration (Fig. S3E); therefore $d_{\text{jam}} \approx 12$ nm is an upper bound. However, because the dynein head/ring diame-

[1]Mean strip length $\bar{L}$ as defined in the main text.

ter (~13 nm) is large relative to a single binding site, such crossings are sterically disfavored. Accordingly, we adopt the stricter jamming criterion

$$d_{\mathrm{jam}} = 12 \text{ nm} \quad \text{(minimum center–axis separation for step acceptance).}$$

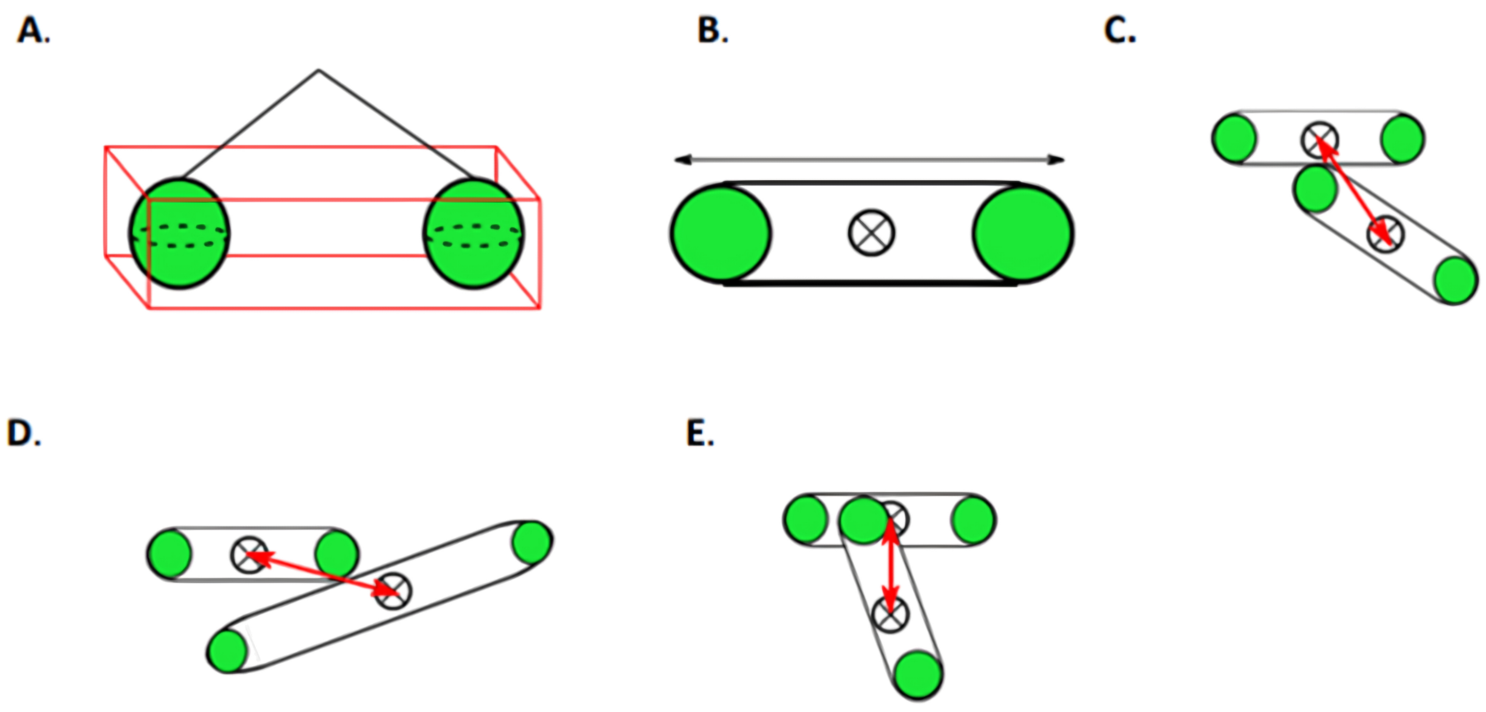

Figure S3.: Forbidden volume and possible configurations for two pairs of binding sites. Binding sites are shown as green circles/spheres; dynein central axes are denoted by ⊗. Red arrows indicate the center–axis separation d_{jam}. (A) Effective volume occupied by a single binding-site pair, including the interstitial region (red square). (B) Overlap of binding-site footprints. (C–D) Examples of two adjacent binding-site pairs at different relative angles α. (E) Crossing configuration of two pairs; this case is excluded from the jamming estimate.

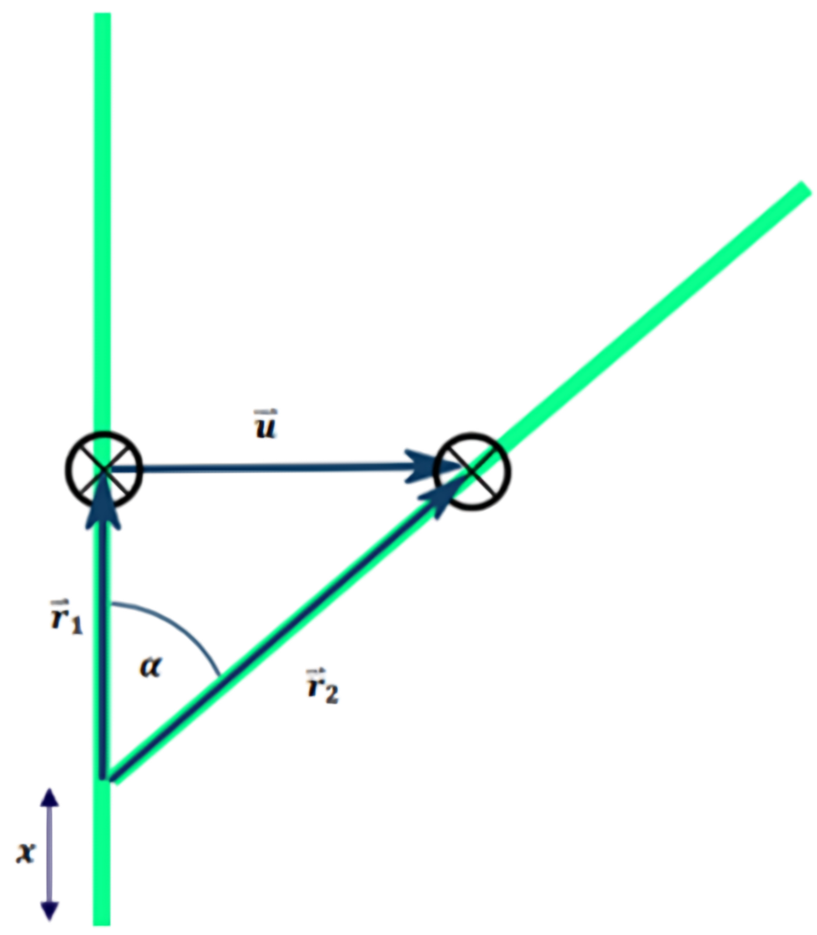

Figure S4.: Schematic configuration of two binding-site pairs. Each pair is shown as a green line segment of length L; dynein central axes are indicated by $\otimes$.

S7. Validation of the Dynein Discretization

To assess numerical convergence of the discrete worm-like-chain representation, we repeated the simulations for six motors using $N = 1, 2, 5, 10, 20$, and 30 equal-length rigid segments per dynein, with $L_p = 2\ \mu$m and 250 nanoparticle trajectories per condition. The mean longitudinal velocity, run-length, and run-time approach a plateau at approximately $N = 10$, indicating that the discretization is sufficient for the simulations reported here. Error bars represent the standard error of the mean.

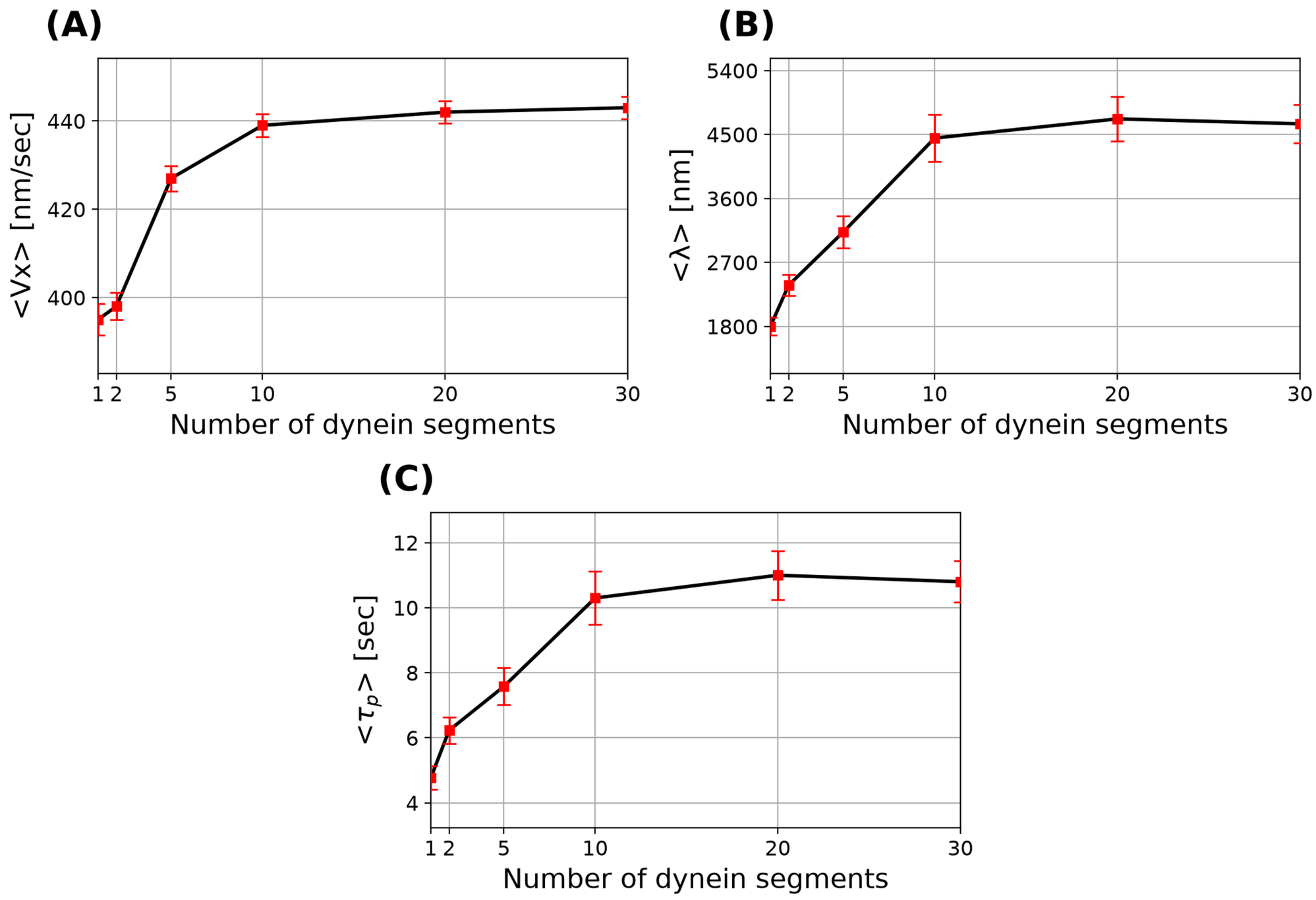

Figure S5.: Numerical convergence of the dynein worm-like-chain discretization. Simulations were performed with six dynein motors grafted to each nanoparticle, 250 nanoparticle trajectories per condition, and $L_p = 2$ μm. The mean (A) longitudinal velocity V_x, (B) run-length λ, and (C) run-time τ_p are shown as functions of the number of equal-length rigid segments N. All three observables approach a plateau at approximately $N = 10$. Error bars represent the standard error of the mean.

S8. Binomial distribution

In laboratory experiments, the number of motors N_m attached to a nanoparticle (NP) is a random variable (not to be confused with motors attached to a microtubule), which is a typical feature of adsorption on a finite-sized surface; thus the number of motors may vary between different particles. However, in simulations, the number of motors is kept constant for each configuration.

To account for these fluctuations, we assume that each site on the nanoparticle surface has an independent adsorption probability θ. This statistical construction is used to represent the experimentally relevant distribution of motor loading rather than as an additional fitted model ingredient. As a result, the number of motors attached to a nanoparticle is distributed according to a binomial distribution:

$$P(N_m; \theta, N) = \binom{N}{N_m} \theta^{N_m} (1-\theta)^{N-N_m}, \tag{S11}$$

where N is the number of available adsorption sites.

However, only particles with $N_m > 1$ can bind to a microtubule, so the conditional

binomial distribution must be considered:

$$P^*(N_m;\theta,N) \;=\; \frac{P(N_m;\theta,N)}{1-(1-\theta)^N}, \qquad N_m \geq 1. \tag{S12}$$

To summarize, the binomial form follows directly from the independent adsorption-site assumption and is not an independently fitted empirical distribution. Conditioning only removes particles that do not enter the MT-bound population that are actively transported by dynein.

Calculating mean variables

Given a value of θ, the average of any variable $f(N_m)$ (e.g., velocity, running time) over particles corresponding to that θ is

$$\langle f(\theta)\rangle \;=\; \sum_{N_m=1}^{N} f(N_m)\,P^*(N_m;\theta,N). \tag{S13}$$

To relate θ to the average number of motors attached to a nanoparticle (taking into account that $N_m > 1$), we use

$$\left\langle N_m^*(\theta)\right\rangle \;=\; \frac{N\,\theta}{1-(1-\theta)^N}. \tag{S14}$$

For each fixed motor number N_m, the reported observable f is estimated from approximately 1000 independent stochastic trajectories as $\bar{f}_{N_m} = N_{\text{traj}}^{-1}\sum_j f_j$, with $\text{SEM}_{N_m} = s_{N_m}/\sqrt{N_{\text{traj}}}$. The approximate 95% confidence interval used in the figures is $\bar{f}_{N_m} \pm 1.96\,\text{SEM}_{N_m}$. For the binomially averaged observables, the conditional probabilities $P^*(N_m)$ are used to weight the mean and the corresponding upper and lower confidence limits separately.

S9. Simulation Flowchart

The simulation pipeline for the nanoparticle–dynein system is summarized in the flowchart in Fig. S6. It outlines initialization, event selection (Gillespie), steric checks (forbidden volume), state updates, and termination criteria.

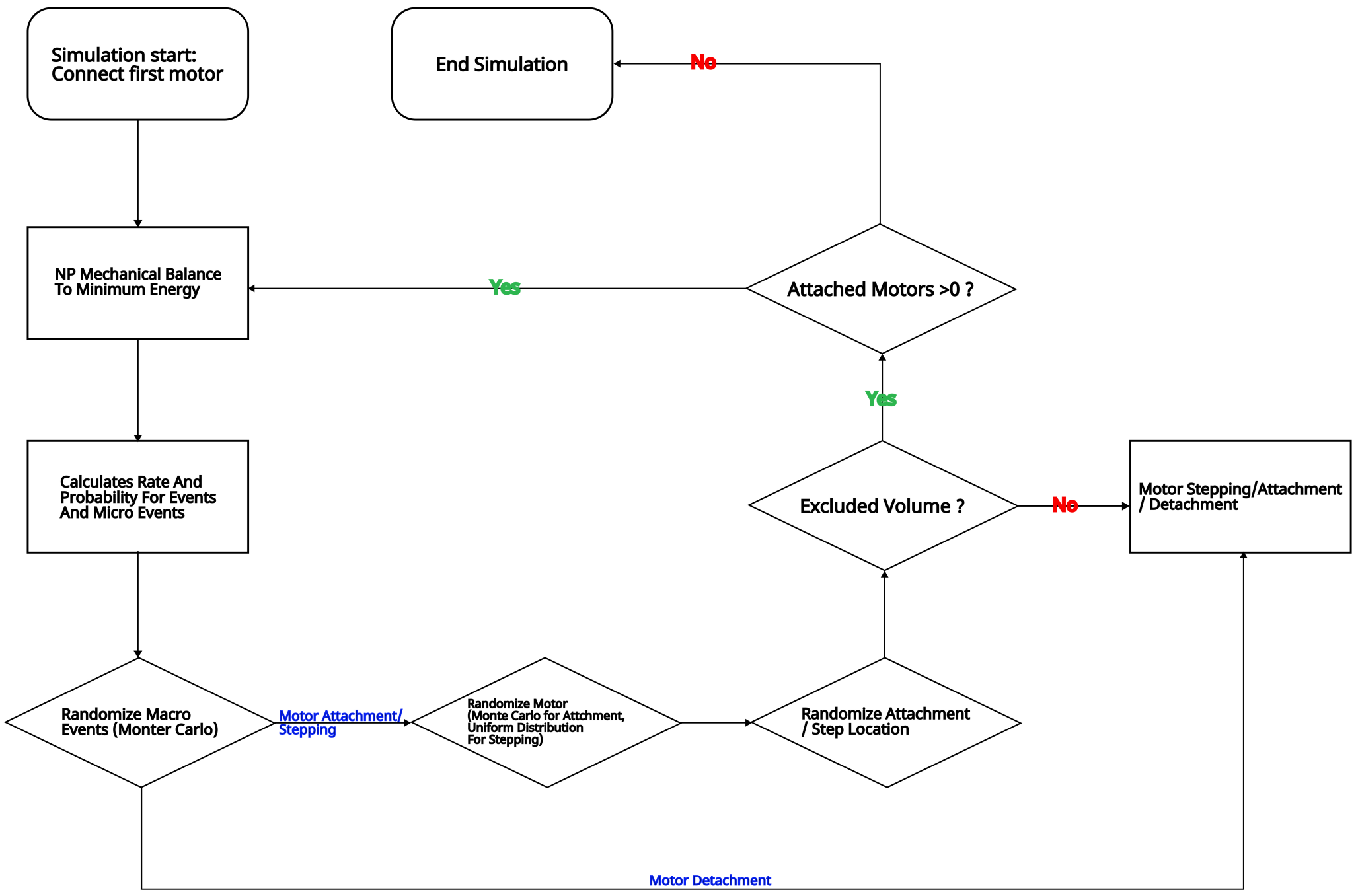

Figure S6.: Simulation flowchart.

References

[1] Oesterhelt F, Rief M, Gaub HE. Single molecule force spectroscopy by afm indicates helical structure of poly(ethylene-glycol) in water. New J Phys. 1999;1:6.1–6.11. Available from: https://doi.org/10.1088/1367-2630/1/1/006.

[2] Lee H, Venable RM, MacKerell J Alexander D, et al. Molecular dynamics studies of polyethylene oxide and polyethylene glycol: hydrodynamic radius and shape anisotropy. Biophys J. 2008;95(4):1590–1599. Available from: https://pubmed.ncbi.nlm.nih.gov/18456821/.

[3] Kienberger F, Pastushenko VP, Kada G, et al. Static and dynamical properties of single poly(ethylene glycol) molecules investigated by force spectroscopy. Single Mol. 2000;1(2):123–128. Available from: https://onlinelibrary.wiley.com/doi/10.1002/1438-5171(200006)1:2%3C123::AID-SIMO123%3E3.0.CO;2-3.

[4] Kon T, Mogami T, Ohkura R, et al. Atp hydrolysis cycle-dependent tail motions in cytoplasmic dynein. Nat Struct Mol Biol. 2005;12:513–519.

Table S3.: **Simulation values chart for semi-flexible/flexible dynein.** Kinematic and dynamical observables obtained from three-dimensional simulations as a function of the number of NP-anchored motors n. Reported uncertainties denote the standard error of the mean (SEM) for τ_p, λ, and V_x. All results are for a 20 nm NP radius and PEG contour length $L_c = 42.56$ nm.

**Semi-flexible (Flexible) Dynein**							
n	τ_p [s]	$\langle\lambda\rangle$ [nm]	V_x [nm/s]	$\langle M_b\rangle$	$\langle\omega\rangle$ [rad/s]	STD(ω) [rad/s]	$\langle H_{\text{kin}}\rangle$ [nm]
1	0.97 ± 0.04	798 ± 37	822 ± 6	1.00	1.58	7.96	2833.74
2	2.40 ± 0.23	1400 ± 113	619 ± 7	1.63	1.42	8.38	2889.61
3	3.50 ± 0.34	1790 ± 157	529 ± 5	1.87	1.28	9.12	2939.74
4	5.27 ± 0.44	2540 ± 202	504 ± 4	2.00	1.18	9.46	2942.71
5	7.39 ± 0.53	3290 ± 228	467 ± 3	2.14	1.08	9.79	3012.16
6	11.5 ± 0.9	4900 ± 356	432 ± 3	2.25	0.97	10.1	3159.28
7	12.4 ± 1.0	5280 ± 417	412 ± 3	2.29	0.90	10.8	3191.30
8	14.7 ± 1.3	5820 ± 475	403 ± 2	2.40	0.86	11.5	3157.60
9	16.0 ± 1.2	6250 ± 452	397 ± 2	2.47	0.82	12.3	3196.95
10	20.7 ± 1.6	7780 ± 570	376 ± 2	2.58	0.78	13.3	3205.57
11	22.0 ± 1.6	7900 ± 565	362 ± 2	2.68	0.75	14.1	3268.02
12	28.1 ± 2.1	10100 ± 714	358 ± 2	2.85	0.72	14.9	3326.31
13	32.1 ± 3.1	11100 ± 814	346 ± 1	2.96	0.70	15.8	3344.34
**Rigid (Non-flexible) Dynein**							
1	1.04 ± 0.03	845 ± 70	843 ± 8	1.00	1.58	7.96	2833.74
2	1.33 ± 0.06	979 ± 80	753 ± 7	1.36	1.23	6.98	3238.13
3	1.99 ± 0.12	1290 ± 101	659 ± 6	1.65	1.08	6.80	3371.47
4	2.41 ± 0.16	1490 ± 115	630 ± 5	1.75	0.96	6.60	3457.97
5	3.74 ± 0.28	2170 ± 163	590 ± 5	1.85	0.86	6.53	3589.31
6	4.72 ± 0.37	2640 ± 196	568 ± 4	1.92	0.79	6.41	3671.53
7	5.57 ± 0.44	3010 ± 222	549 ± 4	1.97	0.74	6.33	3750.27
8	6.45 ± 0.52	3360 ± 246	529 ± 4	2.03	0.65	6.31	3836.59
9	6.87 ± 0.56	3540 ± 259	523 ± 4	2.04	0.60	6.24	3937.35
10	8.79 ± 0.73	4440 ± 322	512 ± 4	2.08	0.56	6.19	4054.57
11	9.39 ± 0.79	4650 ± 337	502 ± 4	2.12	0.51	6.12	4212.23
12	10.8 ± 0.9	5280 ± 381	497 ± 4	2.16	0.47	6.11	4377.86
13	11.4 ± 1.0	5700 ± 410	492 ± 4	2.20	0.44	6.07	4473.34

Table S4.: **Simulation values from variable PEG contour length for semi-flexible and flexible dynein.** Dynamic observables obtained from simulations as a function of (spacer) PEG contour length for semi-flexible (flexible) and rigid (non-flexible) dynein models. All results are for a 20 nm NP radius and $n = 6$ (number of NP-anchored motors.

**Semi-flexible (Flexible) Dynein**			
PEG length [nm]	τ_p [s]	λ [nm]	V_x [nm/s]
10	7.68	2070	299
20	9.02	3130	361
30	9.91	3860	411
40	10.5	4400	432
50	9.49	3990	457
60	8.17	3830	481
70	6.58	3260	504
80	5.80	3250	516
90	5.49	3030	533
**Rigid (Non-flexible) Dynein**			
5	0.963	720	758
7.5	1.99	901	475
10	2.80	910	371
20	5.24	1510	307
30	5.01	1550	328
40	5.50	2050	392
50	4.91	1950	411
60	4.64	2020	454
70	5.45	2500	475
80	4.15	2100	506
90	3.86	1850	520
